# The meaning of wild: Genetic and adaptive consequences from large-scale releases of domestic mallards

**DOI:** 10.1038/s42003-023-05170-w

**Published:** 2023-08-05

**Authors:** Philip Lavretsky, Jonathon E. Mohl, Pär Söderquist, Robert H. S. Kraus, Michael L. Schummer, Joshua I. Brown

**Affiliations:** 1 Department of Biological Sciences, University of Texas at El Paso, El Paso, TX 79668 USA; 2 Department of Mathematical Sciences, University of Texas at El Paso, El Paso, TX 79668 USA; 3 Faculty of Natural Sciences, Kristianstad University, SE- 291 88 Kristianstad, Sweden; 4 Department of Migration, Max Planck Institute of Animal Behavior, 78315 Radolfzell, Germany; 5 Department of Environmental Biology, State University of New York College of Environmental Science and Forestry, Syracuse, NY 13210 USA

**Keywords:** Population genetics, Population genetics

## Abstract

The translocation of individuals around the world is leading to rising incidences of anthropogenic hybridization, particularly between domestic and wild congeners. We apply a landscape genomics approach for thousands of mallard (*Anas platyrhynchos*) samples across continental and island populations to determine the result of over a century of supplementation practices. We establish that a single domestic game-farm mallard breed is the source for contemporary release programs in Eurasia and North America, as well as for established feral populations in New Zealand and Hawaii. In particular, we identify central Europe and eastern North America as epicenters of ongoing anthropogenic hybridization, and conclude that the release of game-farm mallards continues to affect the genetic integrity of wild mallards. Conversely, self-sustaining feral populations in New Zealand and Hawaii not only show strong differentiation from their original stock, but also signatures of local adaptation occurring in less than a half-century since game-farm mallard releases have ceased. We conclude that ‘wild’ is not singular, and that even feral populations are capable of responding to natural processes. Although considered paradoxical to biological conservation, understanding the capacity for wildness among feral and feral admixed populations in human landscapes is critical as such interactions increase in the Anthropocene.

## Abstract (web summary)

A genomic investigation focusing on introgressive hybridization due to anthropogenic causes in mallards reveals a single domestic game-farm breed as the source for contemporary release programs in Eurasia and North America as well as for established feral populations in New Zealand and Hawaii.

## Introduction

An outstanding but evolving question within evolutionary and conservation biology is what are the genetic consequences of hybridization that result from human-mediated disturbances?^1,2^ Unfortunately, with increasing anthropogenic landscape changes, contact between wild and domestic forms seems unavoidable, raising concerns regarding the maladaptive consequences of such interactions on overall fitness and the future adaptive potential of wild populations^1–4^. Alongside changing habitats, domestic releases have become common world-wide, resulting in augmented genetic integrity of various wild populations^5–8^. In fact, in cases where releases have been common and intensive, gene flow from domestic individuals now poses a major threat to these various ecologically and economically important wild species (e.g., rice^9^, trout^10^, salmon^11^, pig^12^, quail^13^, geese^14^, ducks^15–20^, cats^21^, wolves and other wild canids^22,23^). However, genomic data has also revised and refined our understanding where contact between domestic and wild congeners do not always result in wide-spread introgresssive hybridization^24^. More generally, predicting the outcomes of domestic introductions remains challenging because introgression of maladaptive traits into wild populations is additive, and negative consequences are not always immediately apparent^25^. With rapidly changing climatic conditions, it is becoming increasing evident that there is a growing need to understand the genetic underpinning of such events as to predict future challenges in conservation^26–28^. In fact, it is possible that while feral populations are generally unsuited for survival in wild habitats, they may have a greater adaptive potential in human-dominated areas^29^. Here, we employ a landscape genomics approach on global wild and feral mallard (*Anas platyrhynchos*) populations to understand how lineages with varying demographic histories and selection regimes (i.e., natural versus artificial selective pressures) respond to natural versus human influences.

**Fig. 1:**
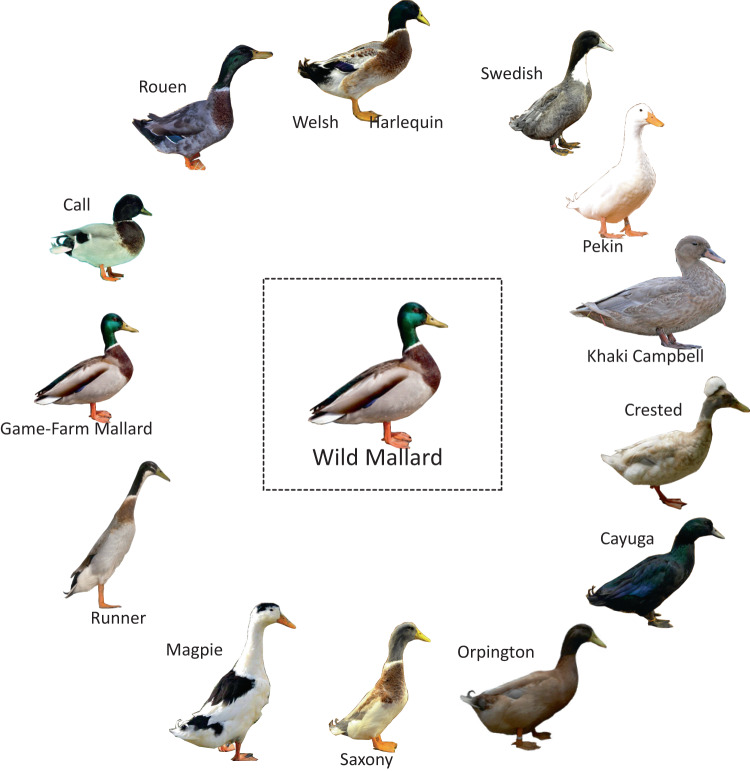
The wild mallard and representation of mallard domestics. Representation of breeds resulting from the domestication of the wild mallard.

Starting in central China, humans have been domesticating mallards since shortly after 500 BC^30,31^. Since then, domestic mallard supplementation has been practiced around the world, with the most intense release programs beginning more recently in the early 20^th^ century^8,32^. While naturally distributed across the Holarctic, the intentional or accidental release of mallards has expanded their range to nearly world-wide outside the Poles^33^. As a result of these efforts, widespread introgression from established feral populations now poses a genetic threat to populations of wild mallards and other closely related waterfowl found throughout Eurasia, North America, and the South Pacific^20,34–37^. Among regions, annual supplementation is currently greatest in Europe and eastern North America where nearly five-million^38,39^ and at least two-hundred thousand (500,000 from 1920s to 1960s;^40–42^) game-farm mallards, respectively, are annually being released. Note that game-farm mallards are captive bred to be released on shooting preserves for hunting and dog training purposes^43^ (Fig. 1). This substantial influx of domesticated individuals has already affected the genetic composition of Eurasian^44^, and North American^16^ wild mallards. In fact, DNA sequencing of historical mallards pre-dating large scale game-farm augmentation events (pre-1920) confirmed that the genetic constitution of today’s eastern North American mallard has been fundamentally altered through extensive gene flow with game-farm mallards^43^.

The [un]intentional release of domestic mallards across regions of varying habitat and ecology provides powerful experiments that can help us to understand how introduced population(s) respond under differing selective pressures, which is an underlying mechanism of local adaptation. We examine the genetic and adaptive consequences from large-scale supplementation practices of domestic mallards by coupling partial genomic sequencing of thousands of samples representing wild and feral populations throughout Eurasia, North America, and New Zealand. After establishing ancestry across wild and feral populations, we couple demographic and phylogenetic analyses to identify the sources of stock currently being released (i.e., in mainland North America and Eurasia) and of two self-sustaining feral populations (Hawaii and New Zealand). Next, we use the program gradient forest to reconstruct the adaptive landscape of wild and feral populations by testing for genotype-environment associations including both natural and anthropogenic indices^45–47^, and thus, reconstructing their genetic niche space^47^. Additionally, we determine whether the association with environmental factors is driven by many or few loci, as well as whether the same loci are consistently under selective pressures from each wild/feral population. In particular, with wild mallards being innately migratory, we predict that associations with environmental cues (e.g., mean diurnal range, temperature, and precipitation) will be consistent across loci for wild populations^47^. Furthermore, we posit that genetic diversity within feral mallard populations that have been shown to take advantage of more human-dominated habitats will be dictated by loci associated with anthropogenic indices (e.g., intensity of urbanization).

## Results

A total of 2951 ddRAD-seq loci (358,999 base-pairs (bp)) were recovered across chromosomes, including 2751 autosomal (333,731 bp), 193 Z-sex chromosome linked (24,331 bp), and 7 W-sex chromosome linked (937 bp) that met our sequencing coverage and missing data criteria for 1916 samples (Supplementary Fig. S1). We obtained an average median depth of 167 sequences/locus, with a median sequencing depth range of 26–235 sequences across samples. Importantly, sex was reliably identified across samples based on sequencing depth ratios of the sex and autosomal chromosomes (Supplementary Data 1 and Supplementary Fig. S2). Finally, a total of 600 overlapping base-pairs of the mtDNA control region were also obtained across the 1862 samples (of 1916); note once again that mtDNA was lacking for the feral mallards of Hawaii (see specifics in Supplementary Data 1).

### Mitochondrial DNA

**Fig. 2:**
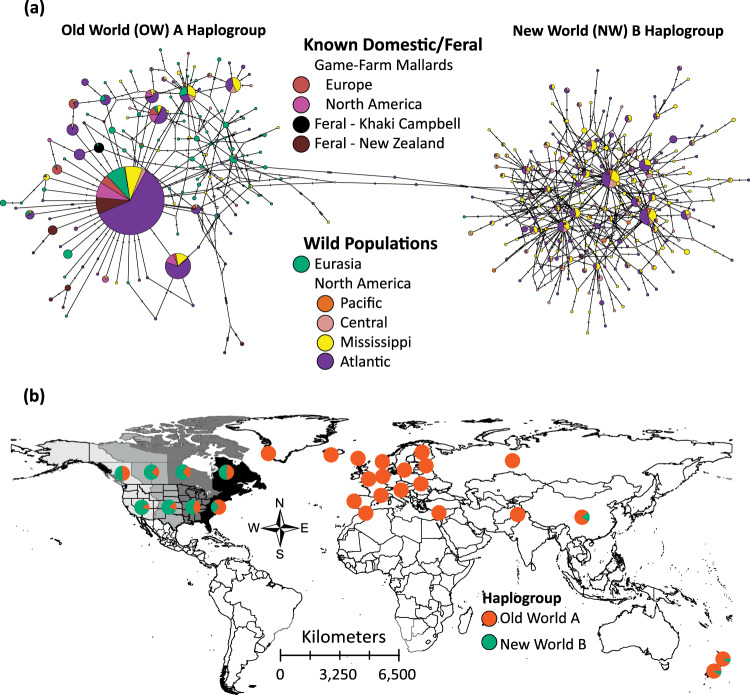
Mitochondrial DNA population structure. **a** A haplotype network based on 600 base-pairs of the mitochondrial control region and sequenced across wild mallards, known game-farm mallards, and feral mallard populations. **b** Plotting the proportion of samples recovered with either the Old World (OW) A or New World (NW) B haplotype across sampled locations. Note that North America was grouped by the four known migratory flyways that waterfowl use during annual migrations.

We recovered known Old World (OW) A and New World (NW) B haplogroups in our mtDNA haplotype network (Fig. 2a;^48–50^). First, all domestic, feral Khaki Campbell’s, and all but one Eurasian wild mallard were recovered within the OW A haplogroup. The one wild mallard from China possessed a NW B haplotype, which may represent a North American migrant; however, being unique, it is also possible that this haplotype represents introgression with endemic spot-billed ducks (*A*. *poecilorhyncha*) that also possess B haplogroup variants^51^. Additionally, a single wild mallard from the Netherlands was recovered within the Khaki Campbell’s haplotype. Although New Zealand’s feral mallards primarily carried OW A haplotypes (91% of samples), eight samples carried six NW B haplotypes not shared with any other wild mallard (Fig. 2a; see Supplementary Data 1 for sample specifics).

In contrast to Eurasian mallards, 56% and 44% of North American wild mallards were recovered in the OW A versus NW B haplogroups, respectively. More importantly, 75% (568 of 791) of all OW A haplotypes recovered in North America were within the same 15 OW A haplotypes carried among sampled game-farm mallards. Similarly, while 13 OW A mtDNA haplotypes were found among New Zealand’s feral mallards, 65% of these samples carried the same major OW A haplotype (Fig. 2a; see Supplementary Data 1 for sample specifics). After accounting for game-farm derived haplotypes, wild mallards within the OW (*N*_*hap*_ = 151) and NW (*N*_*hap*_ = 297) haplogroups showed high uniqueness and variability, whereas game-farm and feral populations showed the complete opposite trend with only 32 haplotypes found across 249 samples (Fig. 2a).

Although plotting the proportion of samples carrying OW A versus NW B haplotypes identified all European countries except one mallard from China as OW A, the presence of OW A mtDNA haplotypes increased eastward in North America^16^ (Fig. 2b).

### Nuclear population structure and phylogenetics

**Fig. 3:**
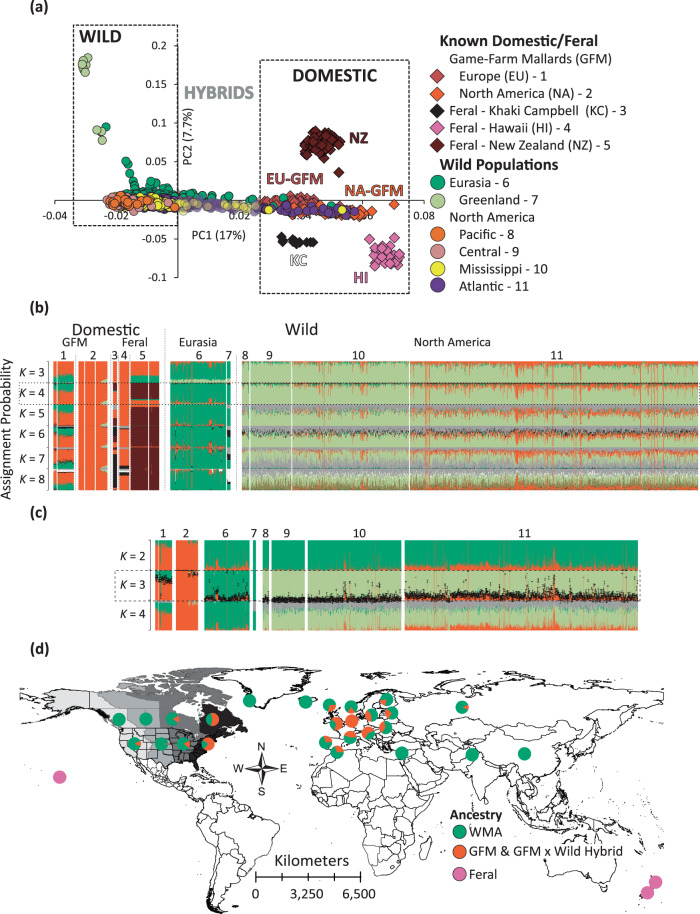
Nuclear population structure analyses. Nuclear population structure analyses based on 36,637 independent bi-allelic nuclear SNPs assayed across 1916 samples comprising wild mallards, known game-farm mallards, and feral mallard populations, and visualized based on a (**a**) Principal Component Analysis, and with ADMIXTURE assignment probabilities obtained for (**b**) all samples or (**c**) just known game-farm mallards and wild mallards from Eurasia and North America. **d** We then plotted the proportion of samples recovered as wild (WMA), GFM-feral /GFM-feral × WMA, or feral across sampled locations. Note that North America was grouped by the four known migratory flyways that waterfowl use during annual migrations.

Population structure analyses were based on 36,637 (of 36,641) independent bi-allelic SNPs. Plotting the first two components of the PCA provided clear groupings of wild and domestic ancestry mallards, with individuals in intermediate space between them as hybrids (Fig. 3a, Supplementary Fig. S3A). Additional separation in PCA space was recovered for wild mallards from Greenland, and for all three of the sampled feral populations (i.e., New Zealand, Hawaii, and Khaki Campbell; Fig. 3a). Next, although an optimum *K* population model of 4 (Supplementary Fig. S3B) was recovered for our ADMIXTURE analysis across all samples, additional resolution was obtained up to a *K* population value of 7 (Fig. 3b). First, we found three wild mallard populations that included Greenland, remaining Eurasia, and North America. Whereas feral populations of Khaki Campbell’s and mallards of New Zealand comprised unique genetic clusters, we confirmed that the game-farm mallard stocks currently being released in Eurasia and North America, as well as the ancestry of Hawaii’s feral population assign to the same genetic cluster (i.e., game-farm mallard genetic cluster; Fig. 3b). Finally, we found that reference game-farm mallards sourced in Eurasia^52^ and from one breeder in North America^53^ have been more recently admixed with wild mallards from their respective regions and consistent with their histories of directed efforts to admix them with wild birds.

Next, because the recovery of all unique genetic clusters in ADMIXTURE required us to evaluate increasing values of *K* populations up to seven, we tested whether ancestry assignments changed among admixed individuals by excluding feral populations. We analyzed known game-farm and wild mallards of Eurasia and mainland North America using a 36,637 independent bi-allelic SNP dataset and following the same protocols in obtaining assignment probabilities from ADMIXTURE as outlined in the methods for *K* population value of 2–4 (Fig. 3c). We again recovered unique genetic structuring between wild mallards of Eurasia and North America, but also found stability in assignment probabilities across samples once interspecific assignments were >10%. In fact, bootstrapped standard-deviations (SD) surrounding individual point estimates to the game-farm mallard genetic cluster was most concerted, averaging ±3%, with a maximum SD of ~6% regardless of which ADMIXTURE dataset was analyzed (Supplementary Fig. S4); and making any sample with ≤6% assignment indistinguishable from pure wild mallard. Conversely, SD values surrounding individual point estimates to wild mallard clusters, particularly as we forced additional population *K* values to be evaluated were much more variable, often deviating by >10% (Supplementary Fig. S4). Thus, we used our ADMIXTURE analysis under a *K* of 3 population model, and assigned individuals as hybrid if they possessed >10% interspecific assignment and all others as pure wild (i.e., assignment probability of <10% to game-farm mallard ancestry) or feral (i.e., assignment probability of ≥90% to game-farm mallard ancestry). We acknowledge that while some samples with assignment probability of 90–95% wild potentially includes late generational backcrosses, we were more concerned as to not create noise in our hybrid sample set by including individuals likely already biologically wild; the same ancestry cutoffs were used in previous studies^16,43,54,55^.

Based on our set ancestry cutoff, only 16 and three North American and Eurasian wild caught samples, respectively, were identified as feral game-farm mallards (Fig. 3d; Supplementary Data 1), representing ~1% of the total sample sets from their respective regions. Plotting the proportion of feral, wild, and hybrid individuals on the landscape established the greatest rates of hybrid prevalence in central Europe and eastern North America, with the proportion of wild ancestry increasing with distance from these areas (Fig. 3d). The westward decline in game-farm × wild mallard hybrid prevalence in North America was concordant with mtDNA patterns (Fig. 2b).

**Fig. 4:**
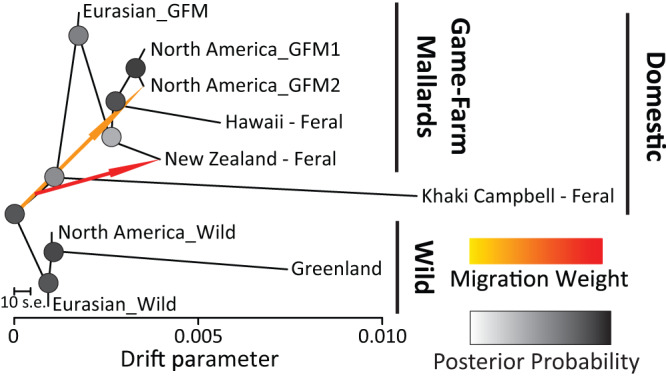
TREEMIX relationships and gene flow events. Relationships among sampled wild, game-farm, and feral mallard groups, as well as gene flow events recovered in tree reconstructions as implemented in the program TreeMix and based on 36,637 independent bi-allelic nuclear SNPs. Grouping of samples were based on respective ADMIXTURE assignment probabilities (Fig. 3; also see Supplementary Data 1). Note that contemporary game-farm × wild hybrids identified in North America and Eurasia were excluded from analyses.

Finally, nine groups were analyzed in TreeMix: (1) three wild mallard genetic clusters including mainland Eurasia, Greenland, and North America, (2) a single domestic game-farm mallard cluster from Eurasia, and two from North America, and (3) feral populations of Khaki Campbell’s, as well as those on Hawaii and New Zealand. An optimum TreeMix tree and >99% of the variance explained was recovered when including up-to two migration edges (Fig. 4; Supplementary Fig. S3C). Two major clades representing wild or domestically-derived groups were recovered. Among these, the Eurasian wild mallard was recovered as basal to all others and consistent with this being the ancestor^30,31^. Next, Khaki Campbell’s were recovered basal to the remaining groups within the domestic clade, which included the Eurasian game-farm mallard basal to North American game-farm mallards and feral populations of Hawaii and New Zealand (Fig. 4). Interestingly, whereas the drift parameter generally increased among groups within the game-farm mallard clade, Khaki Campbell’s and Greenland mallards had comparable and highest drift parameters. Finally, the two statistically significant gene flow events included, gene flow from the wild mallard ancestor to the second North American game-farm mallard group (i.e. GFM 2; Fig. 4), and from the basal domestic lineage into New Zealand mallards; both consistent with their respective mixed supplementation histories^53,56,57^.

### Genomic differentiation and selectively non-neutral loci

**Fig. 5:**
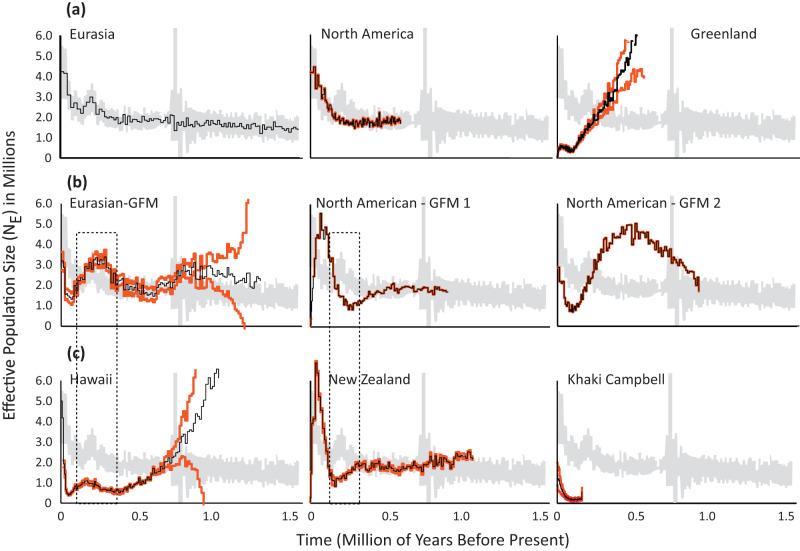
Per species time-series demographic analyses. Per species time-series demographic analyses estimated using the single species ∂a∂i model based on 36,637 independent bi-allelic nuclear SNPs across groups of samples that were partitioned based on their respective ADMIXTURE assignment probabilities (Fig. 3; also see Supplementary Data 1). Contemporary game-farm × wild hybrids identified in North America and Eurasia were excluded from analyses. Eight groups were analyzed: (**a**) wild mallards from Eurasia, North America, and Greenland, (**b**) known game-farm mallards (GFM) Eurasia and North America, as well as (**c**) feral Khaki Campbell and mallard populations on Hawaii and New Zealand. For each of the analyzed groups, we overlay the average (black line) and respective 95% confidence intervals (vermillion lines) over Eurasian wild mallard 95% confidence intervals (in gray) for comparative purposes of estimated effective population size. Boxes denote examples of how differing population histories depress (Hawaii), accentuate positively (Eurasian-GFM), or accentuate negatively (North American –GFM 1 & New Zealand) the ancestral signal.

Genomic comparisons were done on the same nine mallard groups as in phylogenetic analyses (see above). Given that we were interested in general differences across groups, we obtained an average and standard deviation (Φ_ST_ ~ 0.071 ± 0.12) across pair-wise and locus-by-locus comparisons to set an arbitrary two-standard deviation Φ_ST_ threshold of 0.31 for outlier detection (Supplementary Fig. S5). First, composite and locus-by-locus Φ_ST_ estimates recovered little overall genomic differentiation (composite Φ_ST_ ~ 0.004; Supplementary Fig. S5) and no putative outliers when comparing the genomes of wild Eurasian and North American mallards (Fig. 5; Supplementary Fig. S6; Supplementary Data S2). Conversely, Greenland’s wild mallards were most differentiated (composite Φ_ST_ ~ 0.15–0.20; Supplementary Fig. S5A), including highly elevated composite Φ_ST_ estimates with 123 – 670 putative outlier loci across pair-wise comparisons (Supplementary Fig. S6; Supplementary Data S2). While putative outliers were found across the entire genomic landscape of Greenland mallards, 66 loci were recovered across pair-wise comparisons as potential ‘islands of differentiation^58^ (Supplementary Fig. S6; Supplementary Data S2).

Next, composite Φ_ST_ estimates among game-farm mallards recovered little genetic differentiation between the two North American groups (composite Φ_ST_ ~ 0.013), but slightly greater values from Eurasian groups (composite Φ_ST_ ~ 0.03; Supplementary Fig. S5A), including 0–2 putative outliers among pair-wise comparisons (Supplementary Fig. S6; Supplementary Data S2). Finally, when comparing between and within feral lineage, we found fairly consistent composite estimates of relative differentiation respective to each group, with most ranging between 0.05 and 0.20, except for feral Khaki Campbell’s that had consistently high estimates (composite Φ_ST_ > 0.20) across pair-wise comparisons (Supplementary Fig. S5A). The relatively high composite Φ_ST_ estimates of Khaki Campbell’s translated across the genome with >10% of markers identified as putative outliers, including 89 loci spanning the Z-sex and 22 autosomal chromosomes (Supplementary Fig. S6; Supplementary Data S2).

### Genetic diversity & demographics

Similar levels of nucleotide diversity were recovered across groups (average π range = 0.0044–0.0055), except for feral Khaki Campbell’s that had nearly a third of the nucleotide diversity (~0.0016; Supplementary Fig. S5B). Despite having similar levels of genetic diversity, we recovered substantially different demographic histories among groups (Fig. 5). First, near identical demographic histories were recovered for genetically pure wild mallards from North America and Eurasia, which retained an ancestral effective population size of ~1.5 million until ~200,000 years before present when they began to exponentially increase to a contemporary effective population size of ~4.2 million (Fig. 5a). Considering the demographic history of wild mallards as a reference ancestral state, we found the most discordant demographic histories were recovered for Greenland’s wild mallards and feral Khaki Campbell’s (Fig. 5c). Among game-farm mallards, the Eurasian group and the second North American group (i.e., GFM-2) not only possessed exaggerated cyclical demographic patterns in deeper time, but also had a sudden increase in effective population size at time zero (Fig. 5b). Conversely, the North American game-farm mallard that did not show admixed ancestry (Fig. 2; i.e., GFM-1) had exaggerated patterns in more recent time, including a population crash at time zero. Next, New Zealand’s feral mallards had a nearly identical demographic history as North American game-farm mallards, whereas the feral population in Hawaii more closely resembled Greenland’s wild mallards until recently where they experienced substantial growth. Interestingly, despite mallard domestication being initiated around 500 BC^30,31^, demographic deviations from the ancestral state suggests deep time divergence across domestic and feral groups.

### Genetic niche modeling

**Fig. 6:**
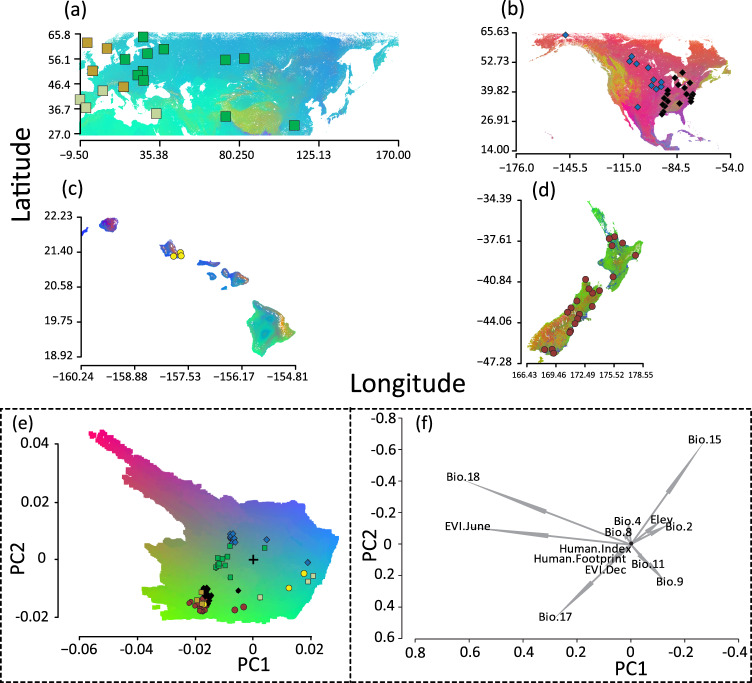
Mapped genotype-environment associations from GRADIENT FOREST analyses. Mapped genotype-environment associations from gradient forest (GF) based on the top five most predictive environmental and/or human index variables (Table 1; Supplementary Fig. S7) across (**a**) Eurasia (wild), (**b**) mainland North America (wild), (**c**) Hawaii (feral), and (**d**) New Zealand (feral). Note that GF models are unitless, and changes in color represent expected changes in allele frequency. Finally, a (**e**) combined GF model overlapping all four groups based on the (**f**) 11 environmental variables and 2 human indices found to be significant in at least one of the four analyses is provided (Table 1). The + in the combined plot denotes the center placement for the PCA of the top predictor variables for reference. Moreover, note that sampling sites for each of the independent analyses are color coded based on their relationship recovered in the combined plot.

The mean R^2^ value for GF models of wild mainland North American (R^2^ = 0.10, *t-test*(62) = 11.19, *p* < 0.001) and Eurasian (R^2^ = 0.15, *t-test*(39) = 2.94, *p* < 0.01), as well as feral populations of mallards in New Zealand (R^2^ = 0.13, *t-test*(99) =  −2.6528, *p* < 0.01) and Hawaii (R^2^ = 0.47; *t-test*(261) = 35.44, *p* < 0.0001) all performed better than their respective randomized datasets (Supplementary Fig. S7). For each group, GF outputs were based on the top five environmental factors with highest adjusted R^2^ values (Table 1; Supplementary Fig. S7). No environmental variable was recovered as significant across all groups, with all but five variables being specific to a single group (Table 1). However, we note that GF recovered a limited accuracy importance (i.e., the out of bag error for GF; Supplementary Fig. S7) for the top environmental variables, suggesting it was unable to parse minute differences between the effects of individual predictors. While this creates some uncertainty with regards to the influence of individual variables, consistency among the top 10 variables identified in individual models suggests that the overall relationship between the environment and genotypic turnover is being reflected. Therefore, in order to account for a lack of more precise differentiation, we included all 13 unique environmental variables (Table 1) when creating the combined GF model (Fig. 6e). Note that GF models were built without human indices for Hawaii’s feral mallards as there was not enough variability among sites for proper analysis (Fig. 6c); however, the combined function of cumulative importance calculated among the other groups were extended to the unknown population sites (i.e., Hawaii; Fig. 6e).

**Table 1:** List and description of the 27 environmental and 2 indices of human impact variables used for genotype-environment association testing in gradient forest analyses.

Variable	Description	Unit	Eur.	NA	HI	NZ
BIO1	Mean annual temperature	°C				
*BIO2*	Mean diurnal range	°C			2	
BIO3	Isothermality	°C				
*BIO4*	Temperature seasonality	°C	2		3	
BIO5	Max temperature of warmest month	°C				
BIO6	Min temperature of coldest month	°C				
BIO7	Temperature annual range	°C				
*BIO8*	Mean temperature of wettest quarter	°C		1		3
*BIO9*	Mean temperature of driest quarter	°C	5			4
BIO10	Mean temperature of warmest quarter	°C				
*BIO11*	Mean temperature of coldest quarter	°C				5
BIO12	Annual precipitation	mm				
BIO13	Precipitation of wettest month	mm				
BIO14	Precipitation of driest month	mm				
*BIO15*	Precipitation seasonality	mm	4		1	
BIO16	Precipitation of wettest quarter	mm				
*BIO17*	Precipitation of driest quarter	mm			4	
*BIO18*	Precipitation of warmest quarter	mm	1	3		
BIO19	Precipitation of coldest quarter	mm				
EVI_ANNUAL	Enhanced Vegetation Index Annual; MOD13A3					
*EVI_SUMMER*	Enhanced Vegetation Index June; MOD13A3				5	
*EVI_WINTER*	Enhanced Vegetation Index December; MOD13A3			5		
NDVI_ANNUAL	Normalized Difference Vegetation Index Annual; MOD13A3					
NDVI_SUMMER	Normalized Difference Vegetation Index June; MOD13A3					
NDVI_WINTER	Normalized Difference Vegetation Index December; MOD13A3					
NPP_ANNUAL	Net Primary Productivity Annual; MOD17A2H					
*SRTM*	Shuttle radar topography mission; Elevation	m	3			
*Human.Footprint*	Anthropogenic footprint; NASA SEDAC data			2		1
*Human.Index*	Anthropogenic index; NASA SEDAC data			4		2

The top five predictive variables based on the cumulative R^2^ weighted importance ranking (Supplementary Fig. S7) used in the respective analysis of wild mallards from Eurasia (Eur.) and North America (NA), as well as feral populations on Hawaii (HI) and New Zealand (NZ) are numerically denoted. The 13 italicized variables were used in the combined analysis (Fig. 6e).

The top five environmental variables for wild mallards in Eurasia included general seasonality in temperature and precipitation, and more specifically, variables associated with average temperature and precipitation during the warmest quarter of the year, and elevation. Although graphing PCs of GF outputs recovered what appeared to be fairly constrained genetic space (Supplementary Fig. S7A), three groups were recovered when plotting genotypic turnover across Eurasia (Fig. 6a), including: (1) Faroe Islands, Britain, Norway, and Slovenia, (2) Russia, Ukraine, Finland, Sweden, Estonia, Pakistan, and China, and (3) regions from France and Spain to Cyprus and Portugal. Interestingly, group one were most distinct from the other two Eurasian groups due to their response to precipitation and anthropogenic influences (Fig. 6e, f).

Next, the top five variables for wild mallards collected during autumn and winter months in North America included weather (i.e., mean temperature during the wettest quarter and precipitation levels during the warmest quarter) and the vegetation index during winter, as well as the human-impact indices (Table 1); graphing PCs of GF outputs showed that wild mallards occupied a fairly diverse adaptive space (Supplementary Fig. S7B). Genotypic turnover was recovered in the northern most arctic regions of Canada, western Rockies, southern regions from Mexico to Florida, and many parts east of the Mississippi River, including northeastern coastal habitat (Fig. 6b). More generally, the primary adaptive range reconstructed from wintering wild mallards recapitulated regions stretching southward from the prairie pothole region of central Canada into the USA, and that are known to be critical migratory and wintering regions for the majority of North America’s wild mallards^59^. In fact, recovery of temperature, precipitation, and vegetative indices as important variables is consistent with other models identifying these as key signals used in migratory initiation in North America^60–62^. Conversely, in the combined GF plot, genotypic turnover became clear between eastern and western North America, explained by the positive or negative association with human-impact indices, respectively (Fig. 6).

Among the two feral populations, GF outputs for New Zealand were based on the average temperature ranges tied to wet-dry cycles, as well as both human indices (Table 1; Supplementary Fig. S7D). Projecting genotypic turnover across New Zealand recovered western parts of the North and South Islands at the edge of the adaptive range for these mallards (Fig. 6d). New Zealand’s mallards clustered with wild mallards of western Europe and eastern North America in the combined GF model; suggesting these groups to be similarly responding to human indices (Fig. 6e, f). Finally, GF analysis of Hawaii’s feral mallards revealed much more reduced genetic space of all groups (Supplementary Fig. S7C), and did not make any predictable clustering in the combined model (Fig. 6e).

## Discussion

### Mallard begets mallard and the proliferation of the game-farm mallard breed

Applying a landscape genomics approach, we establish that genetically pure North American and Eurasian wild mallards are not only structured at mitochondrial DNA as expected (Fig. 2;^50,63,64^), but also identify fine nuclear substructuring (Fig. 3) previously undetected^65^. Although Greenland mallards have previously been found as genetically unique^64,65^, we report previously undetected genetic structure between wild mallards from the rest of Eurasia and North America that is attributable to small frequency differences found across their genomes (Supplementary Fig. S6)^65^. In general, differential patterns of mito-nuclear structure are explained by known life-history traits of mallards that include strong female breeding phylopatry and male-biased dispersal^33^ that often results in strong mtDNA and weak or no nuclear structuring, respectively^48,66^. Despite the population structure, wild Eurasian and North American mallards were generally similar in genetic diversity, with no significant outlier loci detected, and identical demographic histories (Figs. 2–3 and 5; Supplementary Fig. S6). Nevertheless, the strong structuring of Greenland’s mallards suggests that population subdivision has occurred across some parts of the mallard’s Holarctic distribution; but this will require additional sampling to confirm whether Greenland’s mallards diverged through local selective pressures and/or gone through severe genetic drift via founder events.

Next, we provide further evidence that the wild mallard are the primary ancestor of domesticated breeds (Fig. 4;^67–69^), and that game-farm mallards from Eurasia were the ancestral eve of stocks around the world. Whereas wild mallards show a high degree of mtDNA haplotype diversity, samples collected from feral populations and regions where releases are common contain a much smaller set of unique haplotypes (Fig. 2). For example, in eastern North America, 75% of samples with an OW A haplotype were contained within only 15 unique haplotypes. This creates the kind of star-like pattern we recovered in the network (Fig. 2a), which is consistent with a history of game-farm mallard stocks coming from a single or few common lineage(s)^48^. Moreover, we contend that these results clarify the debate about how OW A haplotypes arrived and spread in North America. Whereas past hypotheses suggested more natural pathways for OW A introduction to North America^50,51,70–72^, the geographic distribution and association with game-farm nuclear ancestry strongly support that it was rather a direct consequence of gene flow with introduced game-farm mallards^16^, and thereby anthropogenically induced.

Among attempts at domestication, mallards have been one of the most successful^73^ with over 20 breeds being artificially selected for various uses and functions (Fig. 1). Interestingly, hybridization with domestic breeds, specifically our reference park mallard (i.e., Khaki Campbell’s), is extremely limited, as evidenced by the fact that almost no wild individuals shared any nuclear ancestry with them (Fig. 3). It has become clear, rather, that game-farm mallard releases are the primary pathway of introgression in Eurasia and North America and have already led to self-sustaining and potentially locally adapted feral populations in Hawaii and New Zealand (Fig. 6). In New Zealand, despite releases being discontinued ~70 years ago^34–36^ the self-sustained feral population is now comparable to wild North American mallards in breeding and survival^74^. Moreover, New Zealand mallards seem to be uniquely adapted to their current environment and landscape (Fig. 6e), making them a conservation paradox as their presence remains a major conservation concern for endemic New Zealand grey ducks (*A*. *superciliosa superciliosa*), but are potentially better suited to the increasingly agricultural and urban landscapes of this region (Supplementary Fig. S6;^75–77^). Importantly, our results contrast the general consensus among wildlife managers that game-farm mallards rarely survived long enough to leave the area they are released in, because it is evident from our findings that they survived at great enough rates to have their genetic variation readily found everywhere they were and are currently being released (Figs. 2 and 3;^78^).

Despite opportunities to interact, causes for why domestic × wild mallard hybrids are the result of game-farm mallards and not alternative domestic breeds remains unknown. We posit that it is possible that phenotypic similarities between wild and game-farm mallards may not result in negative assortative mating that may exist between wild mallards and other domestic breeds like Khaki Campbell’s (Fig. 1). Moreover, it is possible that the domestication process results in highly variable genomic changes in extent and type (e.g., chromosomal inversions) that may confer reproductive isolation; but which is evidently absent between wild and game-farm mallards. In fact, Khaki Campbell’s are highly divergent across ddRAD-seq loci as compared to game-farm mallards when compared to wild mallards (Supplementary Fig. S6). Behavioral work and full genome data will be required to test between these respective hypotheses, and in order to better understand potential mechanism(s) that result in variable levels of interactive potential and/or offspring viability that may exist between wild mallards and their various domesticated breeds.

### Sample identity is critical in demographic reconstructions

Given that coalescent events dictate demographic histories^79,80^, we demonstrate how severe loss and the influx of novel genetic diversity via genetic drift (e.g., inbreeding, domestication) and gene flow, respectively, can cause deviations in allelic histories from their true ancestral states, resulting in distorted demographic histories^47,81^. First, we find extreme demographic changes as compared to the wild ancestral state to be among highly bottlenecked groups (i.e., Greenland, Khaki Campbell & Hawaii’s feral mallards; Fig. 5; also see drift values in Fig. 4). Next, the presence and exaggeration of cyclical demographic patterns in deeper time generally corresponds with extent and recency of gene flow (Fig. 5). For example, whereas all the three analyzed game-farm mallard groups showed variable but exaggerated cyclical patterning in deep time, each group either showed a sudden increase (i.e., Eurasian GFM & GFM-2) or crash (i.e., GFM-1) at time zero (Fig. 5b) that followed whether the group experienced recent wild introgression or not, respectively (Fig. 3). In fact, similar patterns of distortion were found in PSMC analyses of full genomes among domestic and wild mallards^68,69^. For example, Guo et al.^67^ inferred domestic lineages to have diverged over 40,000 years ago based on demographic reconstructions, and thus, the domestication of mallards must have been even earlier than expected. However, given that animal husbandry and domestication among human civilizations generally started somewhere between 15,000 and 36,000 years ago with domestication of fowl occurring over the last 5000 years^73,82–84^, we argue that these inferences of demographic history were biased due to distortions created by the severe bottlenecks that occurred among domestic mallard lineages. The same patterning can be observed for game-farm mallards. Although, the true date of mallard propagation remains unknown, raising mallards for sport hunting, and thus the likely rise of the game-farm mallard, was first reported in 1631 England for King Charles II^85^, and the use of game-farm mallard first reported in breeding and ringing operations in 1890s England^86^. Consequently, our demographic results suggesting deep time divergence between game-farm and wild mallards (Fig. 4) is clearly inaccurate based on records indicating that this breed, as it occurs today, ranges from 150 to 400 years old. Overall, we warn that future studies interested in reconstructing demographic histories, which are often used in conservation assessments and decision making, need to be cautious about the individuals they select to reduce bias, especially in lineages that are naturally inbred, domestic, or contain highly admixed individuals^47,81^.

### The conservation paradox of feral populations and the meaning of wild

Manipulating biodiversity through land use change or various human interactions plays an important role across many aspects of society including agriculture, urban development, wildlife management, and nearly all forms of conservation^87,88^; animal domestication in particular, has been essential for the rise of many societies^82–84^. However, the traits favored by artificial selection during domestication can often be deleterious in wild settings, thereby reducing the adaptive potential, fitness, and overall survival of a wild population^8,89–94^. Unfortunately, wild × feral interactions have become more common, and while generally these lead to negative outcomes for the native wild taxa, in some cases, feral populations are able to take advantage and thrive in increasingly urbanized landscapes^29^. Such scenarios could call into question long held conservation priorities; as in, if we are working to preserve biodiversity, should we begin to embrace the potential for introduced feral taxa to thrive in more urban and agricultural habitats, and if so, would this come at the cost or addition to locally adaptive wild diversity? In particular, feral taxa that are already in the focus of conservation efforts that represent ancient lineages with relevant genetic or cultural importance may be perfect cases of naturalization, with the potential of being awarded recognition and protection^95^.

Feral populations are innately different from native wild ones due to their history of artificial selection, and thus, the probability of a group of domestic individuals becoming feral is dependent on their response to the selective pressures of their new environments^27^. Towards this end, the history of domestic mallard releases and established feral populations provides natural experiments that can be used to advance our understanding of ‘feralization’ as a process, as well as species establishment and expansion. First, we determined that the genetic niche space of New Zealand’s feral mallards, as well as wild mallards in Europe and those in eastern North America are explained by precipitation levels during drought years, winter vegetation, and are responding to human disturbances unlike other groups (Supplementary Fig. S7). In fact, maps of genotypic turnover for each of these three groups recapitulate regions in the world with a strong human footprint (Fig. 6a, b, d;^26^). Conversely, the genetic adaptive niche space of wild mallards in eastern Eurasia and western North America, as well as those from France, Spain, and Portugal are largely responding to temperature and precipitation regimes of their regions (Fig. 6). Moreover, we find that within feral populations, New Zealand mallards occupy a unique adaptive space, which could be the result of extensive introgression from locally adapted grey ducks^36^. Overall, these results demonstrate, that in the Anthropocene, the definition of wild may not be singular, and that feral populations are capable of responding relatively quickly to human-induced and natural ecological changes.

While we recovered 1141 and 1360 outlier loci from pair-wise Φ_ST_ and environment association, respectively (Supplementary Figs. S6 and S7), there was a general lack of consistency across analyses; though we found concordance among 23% (750 loci) of recovered putative outlier loci among analyses (Supplementary Data 2). Nevertheless, the lack in Φ_ST_ outlier consistency across respective pair-wise comparisons for wild Eurasian, wild North American, and New Zealand’s feral mallards suggests that experienced selective pressures from their respective niche spaces are differentially impacting parts of their respective genomes. In fact, ~57% of loci found to be significantly associated in GF models were group-specific, suggesting that loci are being independently affected by distinct selective pressures among groups. A lack of overlapping candidate loci is unsurprising given the nature of our ddRAD-seq dataset, which is limited to non-coding regions, meaning that we are only able to detect the effects of selection through hitchhiking^96–98^. Thus, while we find many loci of putatively small effect, identifying a common genetic underpinning of adaptation among these groups will require full genome data and more information regarding protein functions and their response to these various selective pressures^99,100^.

## Conclusions

Going forward, conservation science will need new approaches and practices when contending with challenges presented by climate change and our growing human footprint^100,101^. Mallard introductions in New Zealand and Hawaii provide evidence that feral populations can stabilize and become self-sustaining, as the supplementation and introduction of domestic variants allows adaptive alleles to become prevalent once natural selection becomes a more dominant force. However, it is possible that these feral populations are the product of favorable island conditions where predation and seasonal changes are limited. Thus, we posit that the increasing frequency of game-farm mallard ancestry among wild continental mallards is concerning given the more challenging aspects of migration, predation, and severe seasonal changes. Together, these results beg the question of whether currently declining mallard populations of Eurasia and North America are due to the influx of maladaptive traits from released game-farm mallards, or simply the inability of feral and feral admixed individuals to properly adapt to ecosystems far more complex than captive settings. Determining how domestication changed the morphology, behavior, and/or ecology of game-farm mallards will be required to test whether introgression of such traits is maladaptive for wild populations in ways that result in declining population parameters (e.g., survival and fecundity) and sizes. Regardless, the rising prevalence of feral and non-native populations is becoming a major conservation concern, including being the primary causes of biotic homogenization^102,103^, especially as more endemic species become imperiled in the Anthropocene^29^. Therefore, interaction of wild and domestic animals, as well as the potential establishment of feral or feral admixed populations, require continued monitoring due to the dynamic nature of environmental selective pressures, urbanization, and climate change.

## Methods

### Sampling & DNA extraction

**Fig. 7:**
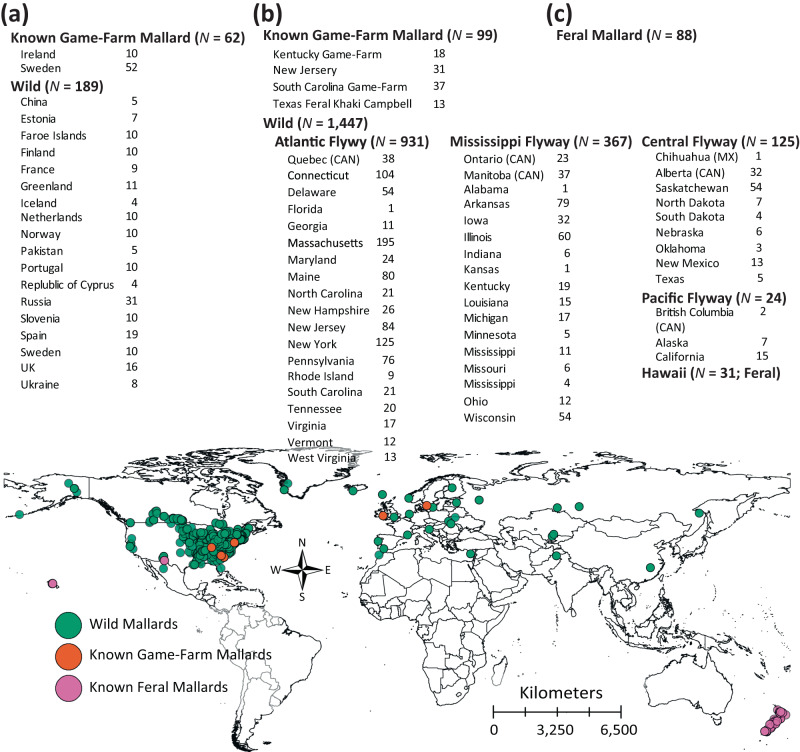
Total and geographical locations of samples. Size and geographical locations of samples obtained across (**a**) Eurasian countries, (**b**) North American states, and (**c**) both New Zealand Islands. Wild mallards, known game-farm mallards, and three feral populations are denoted by color on the map.

Tissue, blood, DNA, or comparable published sequences were obtained for a total of 1916 samples of wild and feral mallard populations representing their ranges in mainland North America, Eurasia, Hawaii, and New Zealand (Fig. 7; Supplementary Data 1). In addition, known domestic stocks of game-farm mallards were sampled from two and three preserves in Eurasia and North America, respectively. Finally, several feral Khaki Campbell (*N* = 13; also see Fig. 1) mallards were also included and served as a proxy of alternative domestic park mallards.

We extracted genomic DNA from blood or tissue using a DNeasy Blood & Tissue kit following the manufacturer’s protocols (Qiagen, Valencia, CA, USA). DNA integrity was based on the presence of a high-molecular weight band as determined with gel electrophoresis using a 1% agarose gel^104^.

### Mitochondrial DNA sequencing & analysis

We PCR amplified and Sanger sequenced the mitochondrial control region (mtDNA) across samples^105,106^ and following protocols outlined in Lavretsky et al.^107^ (also see Supplementary Methods for details). We aligned and edited previously published data for 739 samples and new sequences using Sequencher v. 4.8 (Gene Codes Corporation, Ann Arbor, MI, USA). All new sequences are deposited in GenBank (Accession number: OR089157-OR090779; See Supplementary Data 1 for sample specific information).

Mallards are characterized by the old world (OW) A and new world (NW) B mtDNA haplogroups, which distinguish individuals of Eurasian or North American descent, respectively^48,49,108^. Importantly, being of Eurasian descent, all domestically-derived mallards carry OW A haplotypes, and thus, are a distinguishing marker when assessing whether game-farm mallard introgression likely occurred within a wild mallard lineage in North America^43,109^. Thus, samples were visualized and sorted as possessing OW A versus NW B haplogroups using a median-joining network carried out with the program POPART^110^. Note that although no comparable sequences were available for the same mallards from Hawaii, these were previously shown to possess OW A haplotypes^20,111^. Finally, we plotted proportion of samples with OW A versus NW B haplotypes by European country or North American waterfowl flyway region that generally defines migratory pathways in ArcMap v. 10.7.1 (Esri).

### ddRAD-seq library preparation & bioinformatics

We followed ddRAD-seq library protocols using *Sbf*I and *Eco*RI restriction enzymes, and as outlined in DaCosta and Sorenson^112^^,^(also see ref. ^96^). Size selection followed gel electrophoresis protocols as outlined in DaCosta and Sorenson^112^ for samples collected before 2016, with samples collected thereafter size selected following optimized double-sided magnetic bead selection as described in Hernandez et al.^113^. Because samples were collected over the last decade, multiplexed libraries have been sent to various genomic facilities for 150 base-pair, single-end chemistry sequencing across Illumina platforms including, HiSeq 2000, HiSeq 2500, HiSeq 4000, HiSeq X, and Novoseq. Additional details on DNA extraction and ddRAD-seq library preparation protocols can be found in Supplementary Methods. All raw Illumina sequences are available from the National Center for Biotechnology Information Sequence Read Archive (BioProject accession numbers. PRJNA980669, PRJNA516035^16^, PRJNA911832^114^, PRJNA800412^115^, PRJNA591912^43^, PRJNA847792^47^, PRJNA745366^53^, PRJNA577581^20^, PRJNA718623^116^; Sample specific accession numbers can be found in Supplementary Data 1). In all cases, raw Illumina reads were de-multiplexed using the *ddRADparser.py* script of the BU ddRAD-seq pipeline^112^ based on perfect barcode/index matches.

Across 1916 samples, we used custom in-house Python scripts (Python scripts available at https://github.com/jonmohl/PopGen; see^43^) to automate sequence filtering, alignment, and genotyping using a combination of Trimmomatic^117^, Burrows Wheeler Aligner v. 07.15 bwa;^118^, and Samtools v. 1.7^117^ (detailed methods can be found in Supplementary Methods). Note that reads were aligned to a chromosomal-level reference wild mallard genome^119^. We further filtered VCF files for any base-pair missing >5% of samples that also included a minimum base-pair depth of 5X (i.e., 10X per genotype), and quality per base PHRED scores of ≥30 using VCFtools v. 0.1.15^120^.

Finally, sex was assigned to each sample based on differences in sequencing depth across autosomal and sex chromosome-linked loci^121^. Specifically, for the homogametic sex (i.e., males = ZZ), we expect to find near-zero levels of sequencing depth across W-sex chromosome linked loci but near equal depth for Z-sex chromosome linked loci when compared to autosomal loci. For the heterogametic sex (i.e., females = ZW), we expect to recover about half the sequencing depth at both W- and Z-sex chromosome linked loci as compared to autosomal loci.

### Population structure & phylogenetics

All nuclear population structure was based on independent bi-allelic ddRAD-seq autosomal single nucleotide polymorphisms (SNPs), and without using a priori assignment of individuals to populations or species. The final dataset was obtained by using VCFtools v. 0.1.15^120^ to first extract bi-allelic SNPs, and then PLINK v. 1.9^122^ to filter for singletons, any SNP missing ≥5% of data across samples or in linkage disequilibrium (LD) (detailed methods can be found in Supplementary Methods).

Population structure was first visualized with a Principal Components Analysis (PCA) as implemented in PLINK v. 1.9^122^. Next, assignment probabilities (*Q* values) were estimated with the program ADMIXTURE v. 1.3^123–125^. In addition to obtaining average *Q* values across 100 iterations, standard errors for each analysis were based on 100 bootstrap replicates for each evaluated *K* population model. We evaluated whether the averaged *Q* score and respective standard error overlapped ≥98% population assignment that we considered to represent a genetically pure parental, whereas those individuals assigned to multiple genetic clusters determined to be as hybrids^16^. Detailed methods can be found in Supplementary Methods. We plotted the proportion of samples determined as hybrid or wild by European country and North American waterfowl flyway in ArcMap v. 10.7.1 (Esri).

Next, to reduce the effects of contemporary gene flow events on inferences, we excluded any hybrid identified in population structure analyses (i.e., contemporary hybrids) when estimating relative differentiation (Φ_ST_), nucleotide diversity, and reconstructing phylogenetic relationships of major wild and domestic mallard lineages. To do so, we used recovered genetic clusters from the above population genetics analyses to categorize samples, demarcating those samples that represent parental versus contemporary hybrids. Once done, we estimated pair-wise population relative differentiation (Φ_ST_) and per population nucleotide diversity across ddRAD-seq loci using the PopGenome package in the program R^126^. In addition to composite pair-wise population estimates using concatenated autosomal and Z-chromosome datasets, per-locus estimates of relative differentiation were also estimated across ddRAD-seq loci. A two-standard deviation threshold from the average of Φ_ST_ estimates obtained across pair-wise and locus-by-locus comparisons was used for outlier detection. Although we used a generally arbitrary cutoff, doing so still allowed us to investigate our primary interest of whether the same or different loci showed elevated estimates of relative differentiation across pair-wise comparisons^127,128^.

Finally, the program TreeMix version 1.12^129^ was used to reconstruct and compare evolutionary histories among major wild and domestic mallard lineages. In addition to reconstructing relationships, historical gene flow was also inferred in TreeMix. In short, TreeMix simultaneously estimates a maximum likelihood (ML) species tree, along with the direction and weight (*w*) of gene flow among taxa that best explains analyzed allele frequencies among groups. The optimum number of migration edges was determined by sequentially adding migration events up to 36 (-m 0–36), and then evaluating the proportion of the variance explained by each migration model. Standard errors (-se) were calculated to assess significance among recovered migration edges. In order to limit overconfidence in the tree model, migration edges were added until >99% of the variance in the tree model was explained. Finally, likelihood ratios were calculated using likelihood estimates to assess significance between possible tree models.

### Demographic analyses

Long-term demographic histories of each mallard population was estimated following the approach of Hernandez et al.^113^, which uses ∂a∂i to model changes in effective population size through time based on multi-individual partial genome data. Each species’ SFS was folded and masked before being projected down to account for missing data between groups^113,130,131^. We then estimated confidence intervals (CI) using parameter uncertainty metrics included in ∂a∂i^131,132^. Briefly, ∂a∂i calculates uncertainty values using a Fisher Information Matrix (FIM) that provides a calculation of variance by measuring how much information can be derived from the data with respect to an unknown parameter. We maximized the number of parameters for which ∂a∂i is able to return a true estimate of uncertainty by calculating uncertainty across a range of step sizes (ε = 10^−2^–10^−9^^132,133^; also see detailed methods on uncertainty metrics^113^). ∂a∂i parameters were then transformed into biologically informative values (see Supplementary Methods for details).

### Genotype-environment association modeling with gradient forest

We obtained high resolution (i.e., ~1 km^2^) global environmental data from several public databases, with a focus on 27 annual and seasonal environmental variables shown to have impacts on bird physiology and ecology (Table 1;^47,134^). Specifically, we included 19 climate variables from the worldclim version 1.4 database (https://www.worldclim.org/version1;^134^); Landsat Normalized Difference Vegetation Index (NDVI), Enhanced Vegetation Index (EVI) and Net Primary Productivity (NPP) data from the USGS AppEEARS database (https://lpdaacsvc.cr.usgs.gov/appeears); and elevation data from the Global Land Cover Facility (http://www.landcover.org/).To test the importance of human disturbance on genetic diversity within wild versus feral populations, we additionally downloaded data from the Human Impact Wildlife Conservation Society^135^ and the Human Footprint Wildlife Conservation Society^136^ indices.

Game-farm admixed individuals were excluded from these analyses based on ancestry estimates from ADMIXTURE; additionally, only winter collected samples were used for Eurasia and North America (Hawaii and New Zealand mallards are non-migratory). Following the approach of Bay et al.(also see refs. ^47,137^), we used a genotype-environment association analysis as implemented in the R package gradientForest (GF)^46^. GF analysis was originally created to detect effects of environmental predictor variables on species turnover across a landscape^46^, but has since been adapted for identifying and modeling changes in allele frequency^47,137,138^. In short, we visualized genetic-environmental associations for each of the populations^138^, as well as used the combined GradientForest function to determine differences in genetic niche space among the groups (see ref. ^47^; also see Supplementary Methods for details).

### Statistics and reproducibility

Statistical analyses were performed in either software packages or in R, with run-ready input files deposited in figshare (accession 10.6084/m9.figshare.23396225). This study included a total of 1916 samples, but various subsets of this total were selected to answer more specific questions (also see Supplementary Methods for details).

### Reporting summary

Further information on research design is available in the Nature Portfolio Reporting Summary linked to this article.

### Supplementary information

Supplementary Information-New

Description of Additional Supplementary Files

Supplementary Data 1

Supplementary Data 2

Reporting Summary

## Data Availability

Mitochondrial DNA sequences are deposited in GenBank (Sample specific accession numbers can be found in Supplementary Data 1). All raw Illumina sequences are available from the National Center for Biotechnology Information Sequence Read Archive (BioProject accession numbers. PRJNA980669, PRJNA516035, PRJNA911832, PRJNA800412, PRJNA591912, PRJNA847792, PRJNA745366, PRJNA577581, PRJNA718623; Sample specific accession numbers can be found in Supplementary Data 1). All other source data (e.g. FASTA (Fig. 2), ADMIXTURE input (Fig. 3), TREEMIX input (Fig. 4), ∂a∂i input (Fig. 5), and gradient forest input (Fig. 6) files) used for all figures and tables are also available on Figshare (accession 10.6084/m9.figshare.23396225). All other data are available from the corresponding author (or other sources, as applicable) on reasonable request.
