# Supplementary material for: The meaning of wild: Genetic and adaptive consequences from large-scale releases of domestic mallards
Source: Commun Biol. 2023 Aug 5;6:819. doi: 10.1038/s42003-023-05170-w (PMC10404241; doi:10.1038/s42003-023-05170-w)
Supplement: Supplementary file 1 — Supplementary Information-New [file 42003_2023_5170_MOESM1_ESM.pdf]

Supplementary Material

Supplementary Methods

Sampling & DNA Extraction

Tissue, blood, DNA, or comparable published sequences were obtained for a total of 1,916 samples of wild and feral mallard populations representing their ranges in mainland North America, Eurasia, Hawaii, and New Zealand (Figure 2; Supplementary Data S1). In addition, known domestic stocks of game-farm mallards were sampled from two and three preserves in Eurasia and North America, respectively. Finally, several feral Khaki Campbell ($N = 13$) mallards were also included and served as a proxy of alternative domestic “park mallards”. Genomic DNA was extracted from blood or tissue using a DNeasy Blood & Tissue kit following the manufacturer’s protocols (Qiagen, Valencia, CA, USA). DNA quality was visually assessed on a 1% agarose gel to ensure high molecular weight bands, and quantified using a Qubit 3 Flourometer (Invitrogen, Carlsbad, CA, USA) to ensure a minimum concentration of 20 ng/μL.

Mitochondrial DNA sequencing & analysis

We PCR amplified and Sanger sequenced the mitochondrial control region (mtDNA) across samples. In short, primers L78 and H774 were used to sequence ~655 base pairs of the mtDNA control region [1,2] following protocols outlined in Lavretsky et al. [3]. Final products were sequenced on an ABI 3730 (Applied Biosystems, Life Technologies, Carlsbad, California, USA) machine at the University of Texas at El Paso BBRC Genomic Analysis Core Facility. Sequences were then aligned and edited using SEQUENCHER v. 4.8 (Gene Codes Corporation, Ann Arbor, MI, USA). All new sequences are deposited in GenBank (Accession number: *TBD*; Supplementary Data S1). Note that 739 samples with already published sequences were also included in analyses (see specifics in Supplementary Data S1).

Supplementary Material

Mallards are characterized by the old world (OW) A and new world (NW) B mitochondrial (mtDNA) haplogroups, which distinguish individuals of Eurasian or North American descent, respectively [4-6]. Importantly, being of Eurasian descent, all domestically-derived mallards carry OW A haplotypes, and thus, are a distinguishing marker when assessing whether game-farm mallard introgression occurred within a wild mallard lineage in North America [7,8]. Thus, samples were visualized and sorted as possessing OW A and NW B mtDNA haplogroups using a median-joining haplotype network calculated in the program POPART [9]. Note that although no comparable sequences were available for the same mallards from Hawaii, these were previously shown to possess OW A haplotypes [10,11]. Finally, we plotted proportion of samples with OW A versus NW B haplotypes by European country or North American waterfowl flyway region that generally defines migratory pathways in ArcMap 10.7.1 (Esri).

ddRAD-seq library preparation & bioinformatics

For DNA with high-molecular weight bands and passing concentration, we followed ddRAD-seq library protocols outlined in DaCosta and Sorenson ([12,] also see [13]). In short, genomic DNA was enzymatically fragmented using *Sbf*I and *Eco*RI restriction enzymes, and Illumina TruSeq compatible barcodes ligated for future de-multiplexing. The barcode-ligated fragments were then size selected either by (1) gel electrophoresis (2% low-melt agarose) followed by gel purification using a MinElute gel extraction kit (Qiagen) per manufacturer protocols (see [12,13]), or (2) optimized double-sided bead selection (see [14]). Libraries were then quantified with a Qubit 3 Flourometer (Invitrogen, Carlsbad, CA, USA) and pooled in equimolar amounts. Because samples were collected over the last decade, multiplexed libraries have been sent to various genomic facilities for 150 base-pair, single-end chemistry sequencing across Illumina platforms including, HiSeq 2000, HiSeq 2500, HiSeq 4000, HiSeq X, and Novoseq. In all cases, raw-

illumina reads were de-multiplexed using the *ddRADparser.py* script of the BU ddRAD-seq pipeline [12] based on perfect barcode/index matches.

Across 1,916 samples, sequences were first trimmed or discarded for poor quality using TRIMMOMATIC [15], and then remaining quality reads aligned to a chromosomal-level reference wild mallard genome [16] using the BURROWS WHEELER ALIGNER v. 07.15 (bwa; [17]). Samples were then sorted and indexed in SAMTOOLS v. 1.7 [15] and combined using the *mpileup* function with the following parameters "-c –A -Q 30 -q 30." All steps through *mpileup* were automated using a custom in-house Python script (Python scripts available at https://github.com/jonmohl/PopGen; see [7]). Next, we used VCFTOOLS v. 0.1.15 [18] to filter VCF files for any base-pair missing >5% of samples that also included a minimum base-pair depth of 5X (i.e., 10X per genotype) and quality per base PHRED scores of ≥30.

Finally, sex was assigned to each sample based on differences in sequencing depth across autosomal and sex chromosome-linked loci [19]. Specifically, for the homogametic sex (i.e., males = ZZ), we expect to find near-zero levels of sequencing depth across W-sex chromosome linked loci but near equal depth for Z-sex chromosome linked loci when compared to autosomal loci. For the heterogametic sex (i.e., females = ZW), we expect to recover about half the sequencing depth at both W- and Z-sex chromosome linked loci as compared to autosomal loci.

Population & phylogenetics

All nuclear population structure was based on independent bi-allelic ddRAD-seq autosomal single nucleotide polymorphisms (SNPs), and without using *a priori* assignment of individuals to populations or species. The final dataset was obtained by using VCFTOOLS v. 0.1.15 [18] to first extract bi-allelic SNPs, and then PLINK v1.9 [20] to filter for singletons (i.e., minimum allele frequency (--maf 0.00052), any SNP missing ≥5% of data across samples (--

geno 0.05), as well as any SNPs found to be in linkage disequilibrium (LD) (--indep-pairwise 2 1 0.5). One of the two SNPs was randomly excluded if an LD correlation factor (r^2) > 0.5 was obtained.

Population structure was first visualized with a Principal Components Analysis (PCA) as implemented in PLINK v1.9 [20]. Next, assignment probabilities were estimated with the program ADMIXTURE v. 1.3 [21-23]. For ADMIXTURE, we ran 100 iterations of each *K* model for one through ten populations. The analysis uses a ten-fold cross-validation (CV) with a quasi-Newton algorithm [24] and a block relaxation algorithm for point estimation. Each individual run was terminated once the change in log-likelihood (i.e., delta) of the point estimates increased by <0.0001. The optimal number of populations (*K*) was then based on the lowest averaged CV-error across all 100 replicates per *K*. The package POPHELPER [25] in R was used to convert all ADMIXTURE outputs into CLUMPP v. 1.1 [26] input files. Final assignment probabilities were based on the optimal clustering alignment across all 100 replicates per evaluated population *K* value using the GreedySearch algorithm for 1,000 iterations as implemented in CLUMPP v. 1.1. Moreover, standard errors for each analysis were based on 100 bootstrap replicates. Final individual assignment probability outputs were based on averaging *Q* scores and respective standard errors. Any sample with a Q-score and standard errors overlapping ≥98% population assignment was considered as genetically pure, while those individuals assigned to multiple genetic clusters determined to be as hybrids [27]. We plotted the proportion of samples determined as hybrid or wild by European country and North American waterfowl flyway in ARCMAP 10.7.1 (Esri).

Next, to reduce the effects of contemporary gene flow events on inferences, we excluded any hybrid identified in population structure analyses (i.e., contemporary hybrids) when

estimating relative differentiation (Φ_{ST}), nucleotide diversity, and reconstructing phylogenetic relationships of major wild and domestic mallard lineages. To do so, we used recovered genetic clusters from the above population genetics analyses to categorize samples, demarcating those samples that represent contemporary hybrids. Once done, we estimated pair-wise population relative differentiation (Φ_{ST}) and per population nucleotide diversity across ddRAD-seq loci using the POPGENOME package in the program R [28]. In addition to composite pair-wise population estimates using concatenated Autosomal and Z-chromosome datasets, per-locus estimates of relative differentiation were also estimated across ddRAD-seq loci. A two-standard deviation threshold from the average of Φ_{ST} estimates obtained across pair-wise and locus-by-locus comparisons was used for outlier detection. Although we used a generally arbitrary cutoff, doing so still allowed us to investigate our primary interest of whether the same or different loci showed elevated estimates of relative differentiation across pair-wise comparions [29,30].

The program TREEMIX version 1.12 [31] was used to reconstruct and compare evolutionary histories among major wild and domestic mallard lineages. TREEMIX was also used to test for historical gene flow. In addition to reconstructing relationships, historical gene flow was also inferred in TREEMIX. In short, TREEMIX simultaneously estimates a maximum likelihood (ML) species tree, along with the direction and weight (*w*) of gene flow among taxa that best explains analyzed allele frequencies among groups. Analyses were run across each bi-allelic SNP (-k 1), with global rearrangement occurring during tree building (-global), and with nodal support based on 1,000 bootstraps. The optimum number of migration edges was determined by sequentially adding migration events up to 36 (-m 0 – 36), and then evaluating the proportion of the variance explained by each migration model. Standard errors (-se) were calculated to assess significance among recovered migration edges. In order to limit overconfidence in the tree model, migration

edges were added until >99% of the variance in the tree model was explained. Finally, likelihood ratios were calculated using likelihood estimates to assess significance between possible tree models.

Demographic analyses

Long-term demographic histories of each mallard population was estimated following the approach of Hernandez et al. [14], which uses ∂A∂I to model changes in effective population size through time. We used custom python scripts scripts available at https://github.com/jibrown17/Dove_dadi.demographics; [14] to calculate a one-dimensional site-frequency spectrum (SFS) from Nexus formatted concatenated sequencing data. Each species' SFS was folded and masked [14,32,33] before being projected down to account for missing data between groups ($N_{Wild\ NA}$ = 300 alleles, $N_{NA\ GF1}$ = 100 alleles, $N_{NA\ GF2}$ = 30 alleles, $N_{Wild\ Eurasia}$ = 140 alleles, $N_{Eurasia\ GF}$ = 100 alleles, $N_{Greenland}$ = 18 alleles, N_{NZ} = 130 alleles, N_{HA} = 50 alleles, $N_{Khaki\text{-}Campbell}$ = 14 alleles). Next, based on a custom demographic model ([14]; https://github.com/jibrown17/Dove_dadi.demographics) that uses 100 integration steps, ∂A∂I creates a model SFS that is used to estimate the optimum parameters of effective population ($N_n = v_n$ x N_{Anc}, N_n = effective population size at the nth time interval) and time intervals ($t_n = T_n$ x 2 x N_{Anc} x G, t_n = total years before present at the nth time interval & G = generation time) for each integration step. Optimized parameters are then scaled to the empirical data using θ ($\theta = 4N_{ANC}$ x μ; N_{ANC} = ancestral effective population size), and subsequently used to calculate biologically informative values of effective population size through time [14]. We used the geometric mean calculated across 50 replicates of parameter optimization for each model and estimated the goodness-of-fit for each model by calculating log-likelihood of the model given the empirical data. Finally, we estimated confidence intervals (CI) using parameter uncertainty metrics

included in ∂A∂I [33,34]. Briefly, ∂a∂i calculates uncertainty values using a Fisher Information Matrix (FIM) that provides a calculation of variance by measuring how much information can be derived from the data with respect to an unknown parameter. The FIM requires a step size (ε) to be chosen for the calculation of the numerical derivatives. We note that ∂a∂i is unable to calculate an uncertainty value for a parameter if the numerical derivative of the parameter is negative; therefore, we maximized the number of parameters for which ∂a∂i is able to return a true estimate of uncertainty by calculating uncertainty across a range of step sizes ($\varepsilon = 10^{-2} – 10^{-9}$ [34,35]; also see detailed methods on uncertainty metrics [14]).

Genotype-environment association modelling with gradient forest

We obtained high resolution (i.e., ~1km^2) global environmental data from several public databases, with a focus on 27 annual and seasonal environmental variables shown to have impacts on bird physiology and ecology (Table 1; [36,37]). Specifically, we included 19 climate variables from the worldclim version 1.4 database (https://www.worldclim.org/version1; [37]); Landsat Normalized Difference Vegetation Index (NDVI), Enhanced Vegetation Index (EVI) and Net Primary Productivity (NPP) data from the USGS AppEEARS database (https://lpdaacsvc.cr.usgs.gov/appeears); and elevation data from the Global Land Cover Facility (http://www.landcover.org/).To test the importance of human disturbance on genetic diversity within wild versus feral populations, we additionally downloaded data from the Human Impact Wildlife Conservation Society [38] and the Human Footprint Wildlife Conservation Society [39] indices.

Game-farm admixed individuals were excluded from these analyses based on ancestry estimates from ADMIXTURE; additionally, only winter collected samples were used for Eurasia and North America (Hawaii and New Zealand mallards are non-migratory). Following the

approach of Bay et al.(also see [36,40]), we used a genotype-environment association analysis as implemented in the R package GRADIENTFOREST (GF) [41]. GF analysis was originally created to detect effects of environmental predictor variables on species turnover across a landscape [41] but has since been adapted for identifying and modelling changes in allele frequency [36,40,42]. In short, we visualized genetic-environmental associations for each of the populations [42], as well as used the combined GRADIENTFOREST function to determine differences in genetic niche space among the groups. We used the 'combinedGradientForest' function to account for population structure, as strong intra-specific population structure corresponding to large geographic differences can bias GF models through increased drift and putative bottleneck effects. Briefly, the combine function in R acts to standardize independent models to one another by calculating a combined function of cumulative importance, which represents the overall relationship between allele frequency turnover and the environmental predictor variables among all groups. Additionally, during standardization, cumulative importance functions for each variable are weighted based on the total R^2 value of the individual GF models (see [36]).

GF analyses followed protocols outlined in Brown et al. [36], where we first converted all SNP data into minor allele frequencies, and subsequently filtered any SNP that was polymorphic in fewer than five total sampling sites in Eurasia, North America, and New Zealand or three total sampling sites in Hawaii [42]. To assess the performance of GF models across these populations, we compared 100 models created with randomly generated data to those generated for our empirical data. To identify potential genomic regions under selective pressures, we used SNPs identified as significant by GF to compare across mallard groups. To visualize independent GF models across Eurasia, North America, New Zealand, and Hawaii respectively, values for each of the top five most important environmental variables (based on R^2 weighted importance) were

summarized using a PCA. These transformed values were used to create a RGB color scale to visualize different patterns of adaptive genetic diversity across the landscape. Note that GF figures are created from a PCA of unitless GF values, and are thus a visual representative summary of the genotype-environment association described by the model. Together, change in colors reflect allelic turnover, allowing us to draw conclusions about how the environment has affected genetic diversity and putatively driven adaptation. Finally, we used the combined GRADIENTFOREST function to combine all four independent models of allele frequency turnover to identify differences in genetic niche space between feral and wild groups [36].

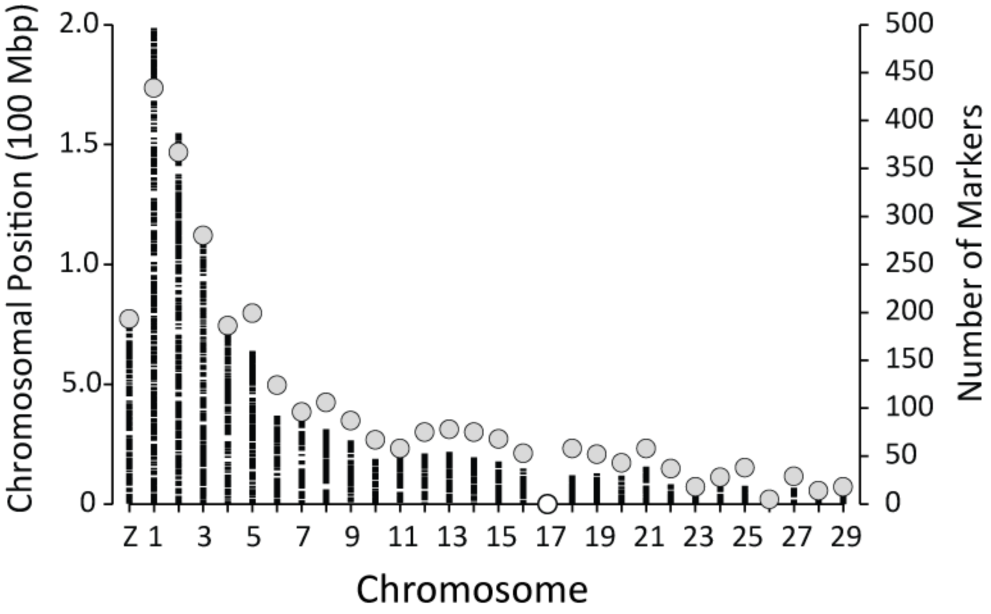

Figure S1. Alignment of 177 Z-linked and 3,017 autosomal ddRAD-seq markers. The Z chromosome is followed by 28 autosomal chromosomes. Grey dots denote the total number of recovered markers per chromosome. Note that no marker was recovered for chromosome 17.

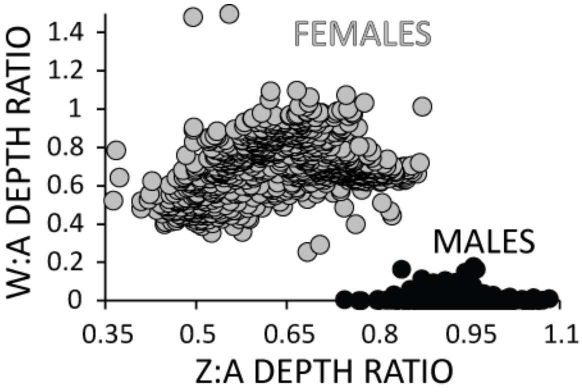

Figure S2. Sex determination based on sequencing depth ratio between ddRAD-seq Z- or W-sex chromosome versus autosomal linked loci.

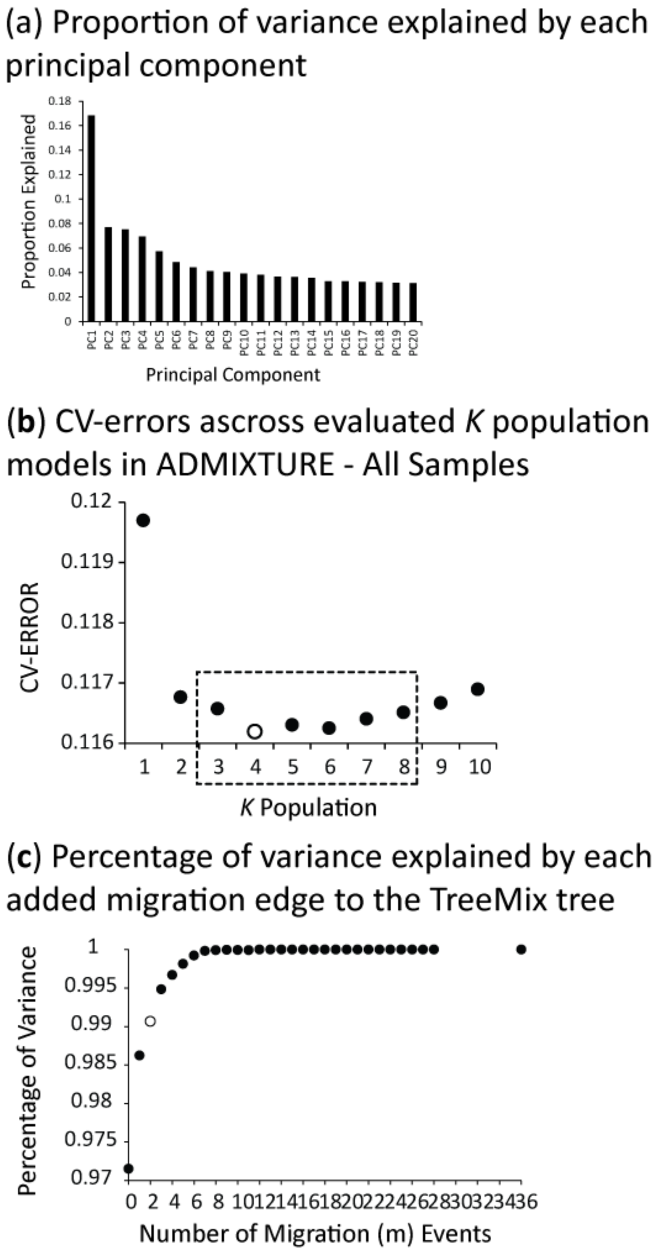

Figure S3. (a) The proportion of variance explained by each principal component in the Principal Component Analysis (Figure 4A). (b) Averaged cross-validation errors across a hundred ADMIXTURE runs per *K* population analyzed using ddRAD-seq autosomal bi-allelic SNPs (Figure 4B). Note that the optimum *K* is in open circle, but all evaluated populations are boxed. (c) The percentage of variance explained with each added migration event to TREEMIX analyses (Figure 5).

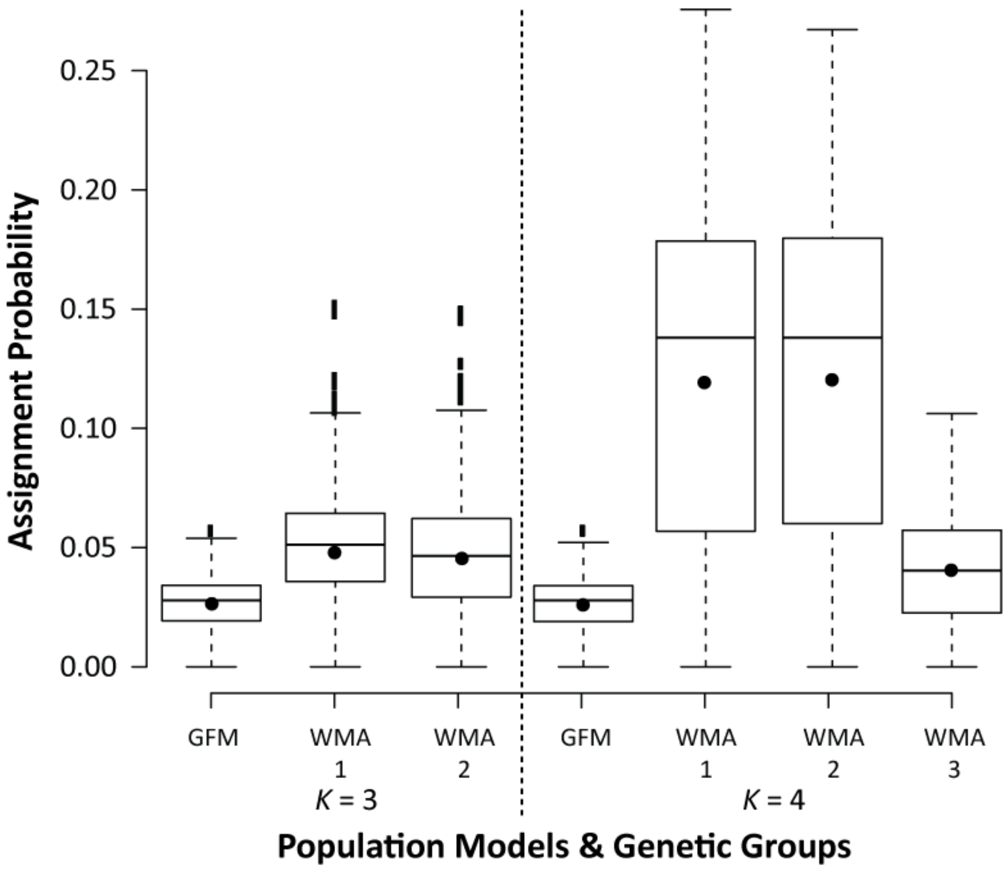

Figure S4. Boxplots and average (black dots) representing the 95% confidence interval of the standard deviations of individual assignment probabilities among genetic clusters recovered from ADMIXTURE analysis of known game-farm mallards (GFM) and wild mallards (WMA) from Eurasia (N_{GFM} = 62; N_{WMA} = 189) and North America (N_{GFM} = 99; N_{WMA} = 1,447) under *K* population models 3 or 4 (Figure 4C). Note that forcing additional population *K* values resulted in further partitioning of the wild mallard, and subsequently causing increased variability in standard deviations as compared to individual assignments to the GFM genetic cluster.

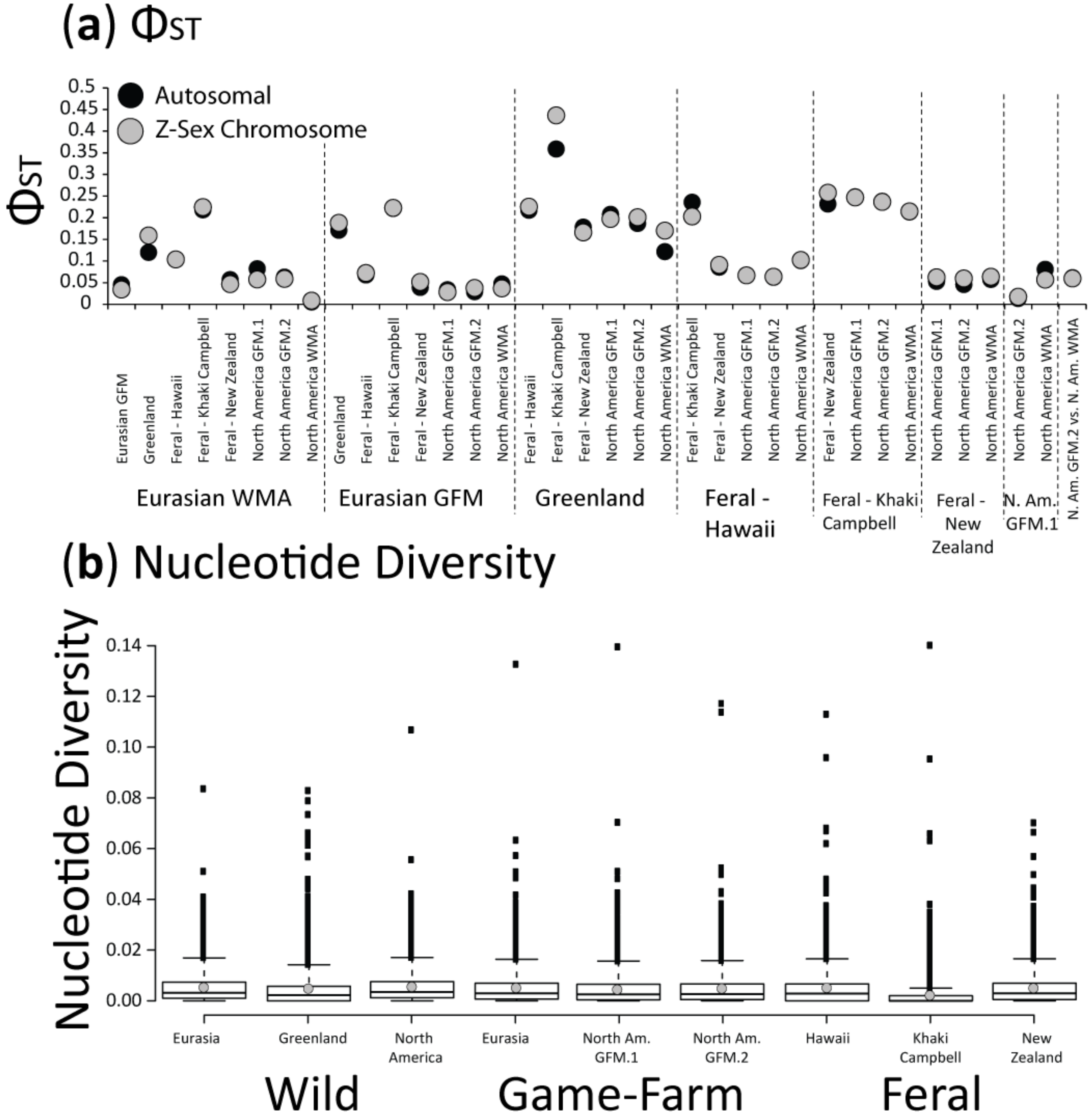

Figure S5. (a) Pair-wise composite relative genetic differentiation (Φ_{ST}) estimates and (b) overall nucleotide diversity across sampled wild (N_{Eurasia} = 118; $N_{\text{Greenland}}$ = 11; $N_{\text{N. America}}$ = 682), game-farm (N_{Eurasia} = 46; $N_{\text{N. America.1}}$ = 67; $N_{\text{N. America.2}}$ = 19), and feral (N_{Hawaii} = 46; $N_{\text{Khaki.Campbell}}$ = 13; $N_{\text{New.Zealand}}$ = 88)mallard groups. Individuals were assigned to each group

based on assignment probabilities (Figure 4C; see sample specific assignments in Supplementary Data S1).

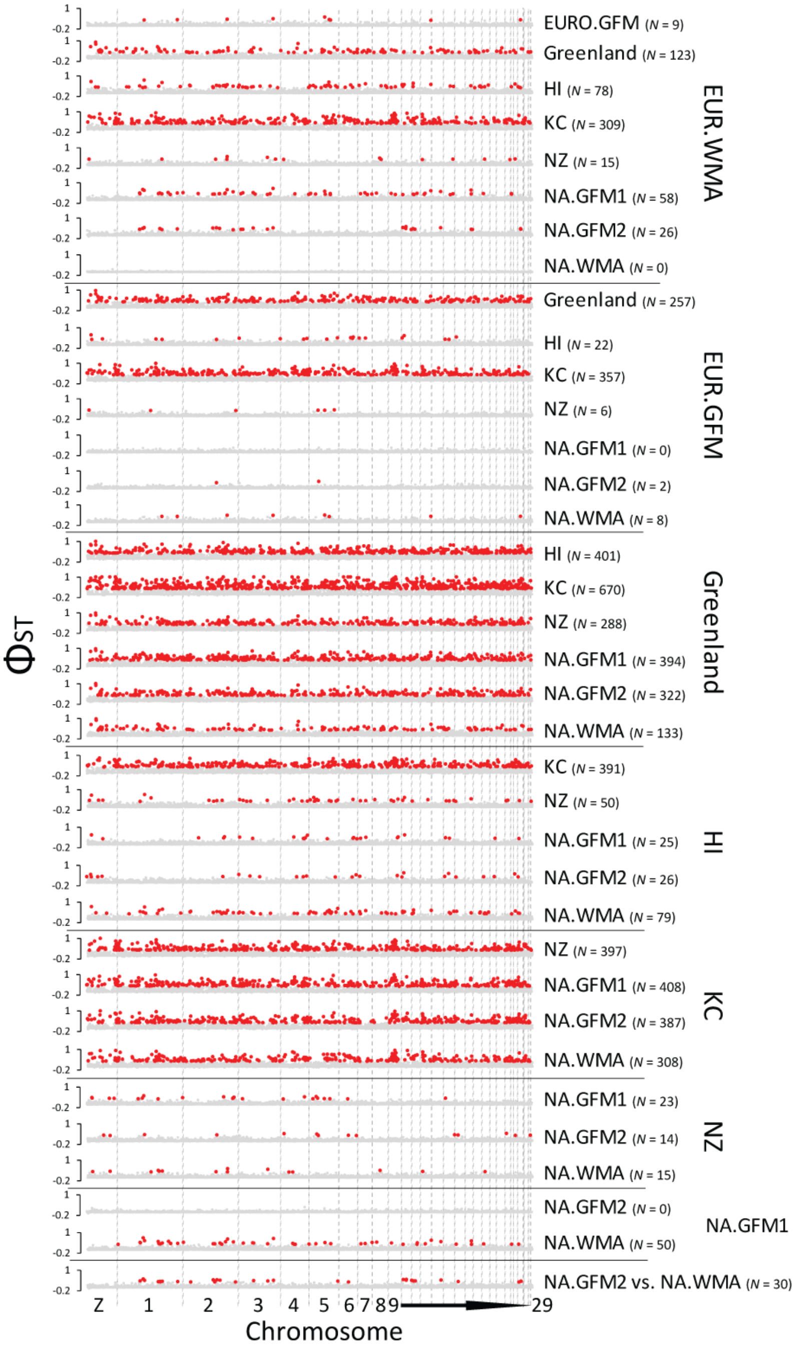

Figure S6. Per ddRAD-seq locus estimates of relative genetic differentiation (Φ_{ST}) across pair-wise population comparisons across sampled wild, game-farm, and feral mallard groups.

Individuals were assigned to each group based on assignment probabilities (Figure 4C; see sample specific assignments in Supplementary Data S1). All putative outlier loci in each respective comparison are red bolded.

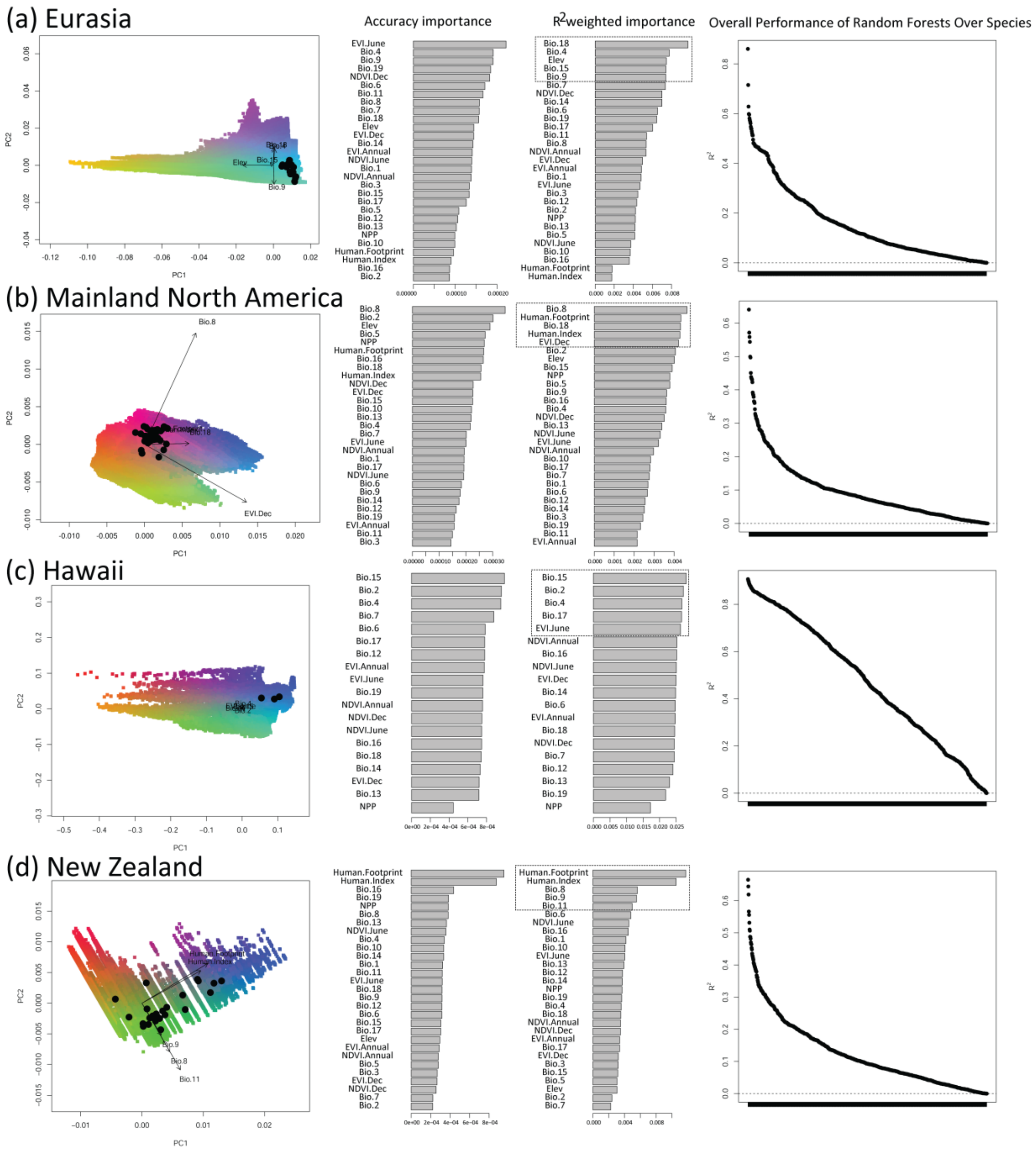

Figure S7. For (a) Eurasian and (b) North American wild mallards, as well as feral populations on (c) Hawaii and (d) New Zealand GRADIENT FOREST (GF) values projected across a PCA of genotype-environment associations based on the top five most predictive environmental and human index variables. Cumulative R^2 weighted values of importance across all analyzed

predictor variables from each groups GF are provided, as well as the R^2 for each SNP identified as significant within the model. Note that GF models are unitless, and changes in color represent expected changes in allele frequency.

Supplementary References

1 Sorenson, M. D., Ast, J. C., Dimcheff, D. E., Yuri, T. & Mindell, D. P. Primers for a PCR-based approach to mitochondrial genome sequencing in birds and other vertebrates. *Molecular Phylogenetics and Evolution* **12**, 105-114 (1999).

2 Sorenson, M. D. & Fleischer, R. C. Multiple independent transpositions of mitochondrial DNA control region sequences to the nucleus. *Proceedings of the National Academy of Sciences of the United States of America* **93**, 15239-15243 (1996).

3 Lavretsky, P., McCracken, K. G. & Peters, J. L. Phylogenetics of a recent radiation in the mallards and allies (Aves: *Anas*): Inferences from a genomic transect and the multispecies coalescent. *Molecular Phylogenetics and Evolution* **70**, 402–411 (2014).

4 Lavretsky, P., Hernández Baños, B. E. & Peters, J. L. Rapid radiation and hybridization contribute to weak differentiation and hinder phylogenetic inferences in the new world mallard complex (*Anas* spp.). *The Auk* **131**, 524–538, doi:10.1642/AUK-13-164.1 (2014).

5 Ankney, C. D., Dennis, D. G., Wishard, L. N. & Seeb, J. E. Low genic variation between black ducks and mallards. *The Auk* **103**, 701-709 (1986).

6 Avise, J. C., Ankney, D. C. & Nelson, W. S. Mitochondrial gene trees and the evolutionary relationship of mallard and black ducks. *Evolution* **44**, 1109-1119 (1990).

7 Lavretsky, P. *et al.* Assessing changes in genomic divergence following a century of human mediated secondary contact among wild and captive-bred ducks. *Molecular Ecology* **29**, 578–595 (2020).

8 Lavretsky, P. in *Population Genomics: Wildlife, Population Genomics* (eds Paul Hohenlohe & Om P. Rajora) (Springer, Cham, 2020).

9 Leigh, J. W. & Bryant, D. POPART: full-feature software for haplotype network construction. *Methods in Ecology and Evolution* **6**, 1110-1116 (2015).

10 Wells, C. P. *et al.* Persistence of an endangered island endemic, an introduced congener, and multiple hybrid swarms across the main Hawaiian Islands. *Molecular Ecology* **28**, 5203-5216 (2019).

11 Fowler, A., Eadie, J. & Engilis, A. Identification of endangered Hawaiian ducks (*Anas wyvilliana*), introduced North American mallards (*A. platyrhynchos*) and their hybrids using multilocus genotypes. *Conservation Genetics* **10**, 1747-1758, doi:10.1007/s10592-008-9778-8 (2009).

12 DaCosta, J. M. & Sorenson, M. D. Amplification biases and consistent recovery of loci in a double-digest RAD-seq protocol. *PloS One* **9**, e106713 (2014).

13 Lavretsky, P. *et al.* Speciation genomics and a role for the Z chromosome in the early stages of divergence between Mexican ducks and mallards. *Molecular Ecology* **24**, 5364–5378, doi:10.1111/mec.13402 (2015).

14 Hernández, F., Brown, J. I., Kaminski, M., Harvey, M. G. & Lavretsky, P. Genomic Evidence for Rare Hybridization and Large Demographic Changes in the Evolutionary Histories of Four North American Dove Species. *Animals* **11**, 2677 (2021).

15 Bolger, A. M., Lohse, M. & Usadel, B. Trimmomatic: a flexible trimmer for Illumina sequence data. *Bioinformatics* **30**, 2114-2120 (2014).

16 Lavretsky, P., Hernández, F., Swale, T. & Mohl, J. E. Genome Report: Chromosomal-level reference genome of a wild North American mallard (Anas platyrhynchos). *G3: Genes,Genomes, and Genetics* (accepted).

17 Li, H. & Durbin, R. Inference of human population history from individual whole-genome sequences. *Nature* **475**, 493 (2011).

18 Danecek, P. *et al.* The variant call format and VCFtools. *Bioinformatics* **27**, 2156-2158 (2011).

19 Lavretsky, P., DaCosta, J. M., Sorenson, M. D., McCracken, K. G. & Peters, J. L. ddRAD-seq data reveal significant genome-wide population structure and divergent genomic regions that distinguish the mallard and close relatives in North America. *Molecular Ecology* **28**, 2594-2609 (2019).

20 Purcell, S. *et al.* PLINK: a tool set for whole-genome association and population-based linkage analyses. *The American Journal of Human Genetics* **81**, 559-575 (2007).

21 Shringarpure, S. S., Bustamante, C. D., Lange, K. & Alexander, D. H. Efficient analysis of large datasets and sex bias with ADMIXTURE. *BMC bioinformatics* **17**, 218 (2016).

22 Alexander, D. H. & Lange, K. Enhancements to the ADMIXTURE algorithm for individual ancestry estimation. *BMC Bioinformatics* **12**, 246 (2011).

23 Alexander, D. H., Novembre, J. & Lange, K. Fast model-based estimation of ancestry in unrelated individuals. *Genome Research* **19**, 1655-1664 (2009).

24 Zhou, H., Alexander, D. & Lange, K. A quasi-Newton acceleration for high-dimensional optimization algorithms. *Statistics and Computing* **21**, 261-273 (2011).

25 Francis, R. M. Pophelper: an R package and web app to analyse and visualize population structure. *Molecular Ecology Resources* **17** 27-32 (2016).

26 Jakobsson, M. & Rosenberg, N. A. CLUMPP: a cluster matching and permutation program for dealing with label switching and multimodality in analysis of population structure. *Bioinformatics* **23**, 1801-1806 (2007).

27 Lavretsky, P., Janzen, T. & McCracken, K. G. Identifying hybrids & the genomics of hybridization: Mallards & American black ducks of eastern North America. *Ecology & Evolution* **9**, 3470–3490 (2019).

28 Pfeifer, B., Wittelsbürger, U., Ramos-Onsins, S. E. & Lercher, M. J. PopGenome: An efficient swiss army knife for population genomic analyses in R. *Molecular Biology and Evolution* **31**, 1929-1936, doi:10.1093/molbev/msu136 (2014).

29 Wolf, J. B. & Ellegren, H. Making sense of genomic islands of differentiation in light of speciation. *Nature Reviews Genetics* **18**, 87 (2017).

30 Campagna, L. *et al.* Repeated divergent selection on pigmentation genes in a rapid finch radiation. *Science advances* **3**, e1602404 (2017).

31 Pickrell, J. K. & Pritchard, J. K. Inference of population splits and mixtures from genome-wide allele frequency data. *PLoS Genetics* **8**, e1002967 (2012).

32 Gutenkunst, R. N., Hernandez, R. D., Williamson, S. H. & Bustamante, C. D. Diffusion Approximations for Demographic Inference: *DaDi*. (2010).

33 Gutenkunst, R. N., Hernandez, R. D., Williamson, S. H. & Bustamante, C. D. Inferring the joint demographic history of multiple populations from multidimensional SNP frequency data. *PLoS genetics* **5**, e1000695 (2009).

34 Coffman, A. J., Hsieh, P. H., Gravel, S. & Gutenkunst, R. N. Computationally efficient composite likelihood statistics for demographic inference. *Molecular biology and evolution* **33**, 591-593 (2016).

35 Blischak, P. D., Barker, M. S. & Gutenkunst, R. N. Inferring the demographic history of inbred species from genome-wide SNP frequency data. *Molecular biology and evolution* **37**, 2124-2136 (2020).

36 Brown, J. I., Harrigan, R. J. & Lavretsky, P. Evolutionary and Ecological Drivers of Local Adaptation and Speciation in a North American Avian Species Complex. *Molecular Ecology* **31**, 2578-2593 (2022).

37 Hijmans, R. J., Cameron, S. E., Parra, J. L., Jones, P. G. & Jarvis, A. Very high resolution interpolated climate surfaces for global land areas. *International Journal of Climatology: A Journal of the Royal Meteorological Society* **25**, 1965-1978 (2005).

38 Wildlife Conservation Society - WCS & Center for International Earth Science Information Network - CIESIN - Columbia University. (NASA Socioeconomic Data and Applications Center (SEDAC), Palisades, New York, 2005).

39 Wildlife Conservation Society - WCS & Center for International Earth Science Information Network - CIESIN - Columbia University. (NASA Socioeconomic Data and Applications Center (SEDAC), Palisades, New York, 2005).

40 Bay, R. A. *et al.* Genomic signals of selection predict climate-driven population declines in a migratory bird. *Science* **359**, 83-86 (2018).

41 Ellis, N., Smith, S. J. & Pitcher, C. R. Gradient forests: calculating importance gradients on physical predictors. *Ecology* **93**, 156-168 (2012).

42 Fitzpatrick, M. C. & Keller, S. R. Ecological genomics meets community-level modelling of biodiversity: Mapping the genomic landscape of current and future environmental adaptation. *Ecology letters* **18**, 1-16 (2015).
