# Supplementary material for: The meaning of wild: Genetic and adaptive consequences from large-scale releases of domestic mallards
Source: Commun Biol. 2023 Aug 5;6:819. doi: 10.1038/s42003-023-05170-w (PMC10404241; doi:10.1038/s42003-023-05170-w)
Supplement: Supplementary file 2 — Description of Additional Supplementary Files [file 42003_2023_5170_MOESM2_ESM.pdf]

Description of Additional Supplementary Files

File name: Supplementary Data 1
Description: Individual sample and locale information.

File name: Supplementary Data 2
Description: Outlier ddRAD-seq locus information.
